# Inside the Mind of a Medicinal Chemist: The Role of Human Bias in Compound Prioritization during Drug Discovery

**DOI:** 10.1371/journal.pone.0048476

**Published:** 2012-11-21

**Authors:** Peter S. Kutchukian, Nadya Y. Vasilyeva, Jordan Xu, Mika K. Lindvall, Michael P. Dillon, Meir Glick, John D. Coley, Natasja Brooijmans

**Affiliations:** 1 Center for Proteomic Chemistry, Novartis Institutes for BioMedical Research, Cambridge, Massachusetts, United States of America; 2 Department of Psychology, Northeastern University, Boston, Massachusetts, United States of America; 3 Global Discovery Chemistry, Novartis Institutes for BioMedical Research, Emeryville, California, United States of America; 4 Blueprint Medicines, Cambridge, Massachusetts, United States of America; University of Alabama at Birmingham, United States of America

## Abstract

Medicinal chemists’ “intuition” is critical for success in modern drug discovery. Early in the discovery process, chemists select a subset of compounds for further research, often from many viable candidates. These decisions determine the success of a discovery campaign, and ultimately what kind of drugs are developed and marketed to the public. Surprisingly little is known about the cognitive aspects of chemists’ decision-making when they prioritize compounds. We investigate 1) how and to what extent chemists simplify the problem of identifying promising compounds, 2) whether chemists agree with each other about the criteria used for such decisions, and 3) how accurately chemists report the criteria they use for these decisions. Chemists were surveyed and asked to select chemical fragments that they would be willing to develop into a lead compound from a set of ∼4,000 available fragments. Based on each chemist’s selections, computational classifiers were built to model each chemist’s selection strategy. Results suggest that chemists greatly simplified the problem, typically using only 1–2 of many possible parameters when making their selections. Although chemists tended to use the same parameters to select compounds, differing value preferences for these parameters led to an overall lack of consensus in compound selections. Moreover, what little agreement there was among the chemists was largely in what fragments were *undesirable*. Furthermore, chemists were often unaware of the parameters (such as compound size) which were statistically significant in their selections, and overestimated the number of parameters they employed. A critical evaluation of the problem space faced by medicinal chemists and cognitive models of categorization were especially useful in understanding the low consensus between chemists.

## Introduction

A core function of human cognition is to reduce the complexity of the world to manageable proportions. In everyday life, we ignore most of the information available in the environment in an attempt to focus on what is likely to be most important. In some professional contexts, this process is raised to an art form, providing a useful context in which to investigate the human cognitive response to complexity.

For instance, in research departments across the pharmaceutical industry, medicinal chemists routinely sift through long lists of compounds with associated data (biochemical activities, physicochemical properties, etc.) in order to prioritize some for further optimization or study, and discard others in the search for new drug candidates. [1] Although computational tools have been developed to aid compound prioritization, [2] medicinal chemists remain intimately involved in compound review. In order to prioritize compounds, chemists must consider whether they possess desirable physical chemical properties (e.g., solubility), how easily they can be synthetically accessed and chemically manipulated, and whether they can be optimized to bind a desired target while avoiding undesirable biological properties such as off-target interactions or mutagenicity. Indeed, guiding compounds through all the potential pitfalls that lie between an initial ensemble of hits and a drug candidate is an extremely complex task, and the selection of the initial chemical starting points for this endeavor greatly impacts the path that is explored, and the ultimate success of a drug discovery campaign.

In this paper we examine how chemists tackle this problem as a way of addressing the more general question of how humans deal with cognitive complexity. Specifically, we asked chemists to sort through ∼4,000 chemical fragments over several sessions, and to identify those they deemed attractive for follow-up. (Chemical fragments are compounds with molecular weight<300, that are smaller than typical drug-sized compounds. They are used as starting points for building larger, more drug-like compounds.) We built classification models to best characterize which objective properties of the fragments were most predictive of each individual chemist’s decisions. In order to ascertain the potentially complex patterns of features that chemists might find desirable or undesirable, we applied two orthogonal classification algorithms: semi-naïve Bayesian (SNB) and Random Forest (RF). While both methods are capable of identifying important features and recognizing complex interdependencies between features, SNB is more readily interpretable. Thus both methods were used to identify important features, while SNB models were used to visualize and interpret chemists’ preferences. We also asked chemists to explain their decision-making. We aim to address three major questions: 1) How and to what extent do chemists simplify the problem of identifying promising chemical fragments to move forward in the discovery process? 2) Do different chemists use the same criteria for such decisions? 3) Can chemists accurately report the criteria they use for such decisions? Below we provide a background for these three questions.

### Reducing Complexity

For most decisions we face in the real world based on sampling available information, the world is much like a superstore – it offers too much, and most of what’s offered does not meet our specific requirements. Given this state of perpetual information overload, people are bound to filter out a great deal of information. Classic work in cognitive science has been critical of this strategy, portraying human reasoning as plagued with biases, based on heuristics that ignore relevant information, and prone to fallacies. [4], [5] This work claims that cognitive limitations lead people to selectively attend to a subset of available information and therefore to systematically make non-normative decisions.

However, recent developments in the study of reasoning question the idea that “less” always means “worse.” As Gigerenzer, Todd, and the ABC research group proposed, [6] the accuracy-effort trade off is not the only reason why people resort to using incomplete information. In certain environments (i.e., those characterized by high cue redundancy [a cue can be thought of as a feature that signals something. For example, shorts and cleats are cues that someone is a soccer player], low predictability of outcomes, or with a small amount of evidence relative to the number of potentially available cues), heuristic-based reasoning that efficiently ignores some of the available information and uses simpler computations can in fact lead to more accurate decisions. [8] In one study, the predictive accuracy of two relatively simply heuristics–“tallying” and “take-the-best”–was compared to multiple regression, a more complex estimation technique, in 20 scenarios ranging from predicting fish fertility to fuel consumption. [10] (The tallying heuristic ignores cue weights and simply counts the number of favoring cues, while take-the-best searches through cues in order of validity and bases a decision on the first cue that discriminates between the alternatives. Regression methods weight the cues differentially, and uses all of them when making predictions.) Regression was shown to be superior in fitting the available data, but its flexibility came with the price of capturing unsystematic patterns in the data, and it was ultimately outperformed by both heuristic methods when it came to prediction (see also [11]). Such “less-is-more” effects - where less information leads to higher accuracy - have been observed in a variety of settings. For example, expert sports players often make better decisions under time pressure. [12], [13] It appears that for some kinds of problems and environments, ignoring pieces of available information can be a signature of expert decision making rather than faulty reasoning.

Consistent with this view, experts often use only a subset of available information in decision making. This has been observed in fields as diverse as medical radiology, [14] medical pathology, [15] stock trading, [16], clinical psychology, [17] and grain judging. [18]–[20] Moreover, experts appear to utilize fewer cues in realistic decision-making settings than in more controlled experimental settings. [21] For example, judges tended to use all available information when reaching decisions in a simulated courtroom setting, but only a small subset in an actual courtroom. [22] Indeed, experts do not appear to differ from novices in the *amount* of information they use, but rather *what* information they use, suggesting that experts are more capable of discriminating what is diagnostic from what is not [23].

In this paper, we address the question of how expert medicinal chemists approach the problem of selecting promising compounds from large sets. Do they aim for exhaustive assessment of each compound, by taking into account all pieces of available information, or do they simplify the problem by focusing on a small subset of compound properties?

### Consensus among Experts

Another question of interest is the degree to which highly-trained and experienced medicinal chemists agree with each other when making decisions about promising chemical fragments. In a seminal paper, Einhorn argued that consensus among experts is a mark of expertise, implying that a lack of consensus among experts demonstrates a lack of expertise. [15] However, evidence from previous work on expert agreement is mixed. First, consensus proved to vary with the domain of expertise [24]: for example, stockbrokers have demonstrated low consensus, [16] while weather forecasters have demonstrated high consensus. [25] Shanteau proposed that the degree of consensus among experts may depend on the properties of the *problem space*, such as predictability [24], [26].

Second, prior work on expert classification suggests that *expert specialization* can affect consensus within a common domain of expertise. For instance, tree experts with different specializations (maintenance, landscaping, or taxonomy) overall agreed in their classification of local tree species, but only landscaping experts showed a distinct tendency to group trees based on their utilitarian value. [27] Similarly, a comparison of Native American and majority-culture fisherman in northern Wisconsin showed overall consensus in their categorization of local freshwater fish species, but also clear differences with respect to the use of morphological (majority-culture) and ecological (Native American) dimensions [28].

Turning to our domain of interest, medicinal chemistry, reports of consensus between chemists from previous studies have been varied. When assessing the synthetic accessibility of compounds, chemists have demonstrated both a considerable amount of consensus (the correlation coefficient *r* ^2^ between chemists ranged from 0.73 to 0.84), [29] and moderate consensus (*r* ^2^ ranged from 0.50 to 0.63). [30] Lower consensus was observed when chemists assessed the drug-likeness of compounds (*r* ^2^ ranged from 0.40 to 0.56). [30] In a study most relevant to the current paper, chemists asked to remove undesirable compounds from lists of putative compounds for inventory acquisition showed little consensus. [31] One difference in the present work is that in our case chemists were asked to actively select desirable compounds, rather than reject undesirable compounds. More importantly, we have gone a step further by analyzing what criteria individual chemists use to select desirable compounds, revealing *why* there is an apparent lack of consensus, and the degree – if any – to which these criteria are consistent across chemists.

### Expert Awareness of Decision Criteria

The counsel of experts is often sought on subjects or items within their field that are too complex for a non-expert to handle – for example, bloodstock agents are consulted to assess how promising a yearling thoroughbred horse is prior to purchase, or a specialized doctor might be sought to diagnose a puzzling symptom. These assessments are often summarized in verbal or written reports, which in turn inform decisions. It would seem almost ludicrous for an expert to make an important recommendation based on their “gut feeling,” yet there seems to be mounting evidence that the unconscious mind under certain circumstances in fact outperforms the conscious mind. Research suggests that the unconscious is especially good at making complex decisions, [32] and that introspection can actually reduce the quality of decisions. [33] It has also been reported that humans are often unaware of the important factors that play a role during complex problem solving. [34] Furthermore, people seem to be ultimately less satisfied with choices that were consciously made, compared to those made unconsciously. [35], [36] Importantly, complex pattern recognition, which is especially relevant to the current study, can be obtained unconsciously. [37] This invites one to reconsider the role of the conscious and unconscious mind when expert chemists prioritize compounds. When faced with the inherently complex problem of assessing the desirability of a compound, are chemists aware of the criteria they use when selecting compounds to carry forward during drug discovery campaigns?

## Results

### Reducing Complexity

Chemists (N = 19) were asked to select desirable fragments from 8 batches of 500 fragments each. In order to determine the number and type of properties that best predicted each chemist’s decisions, we built semi-naïve Bayesian (SNB) and Random Forest (RF) classifiers based on individual chemist’s selections. Medicinal chemistry relevant descriptors were used to train the classifiers, so that the resulting models could readily be related to what types of information (or parameters) were important during selections.

**Figure 1:**
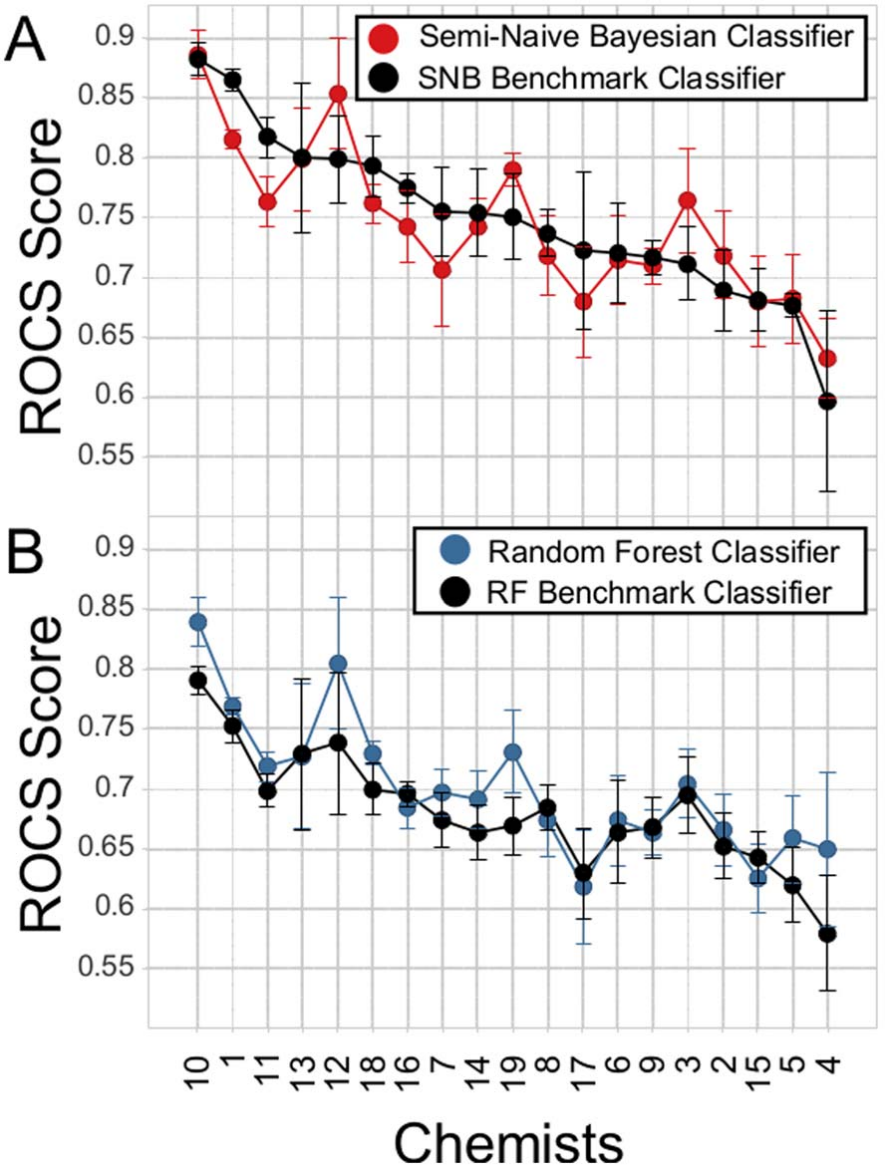
Predictive accuracy of Semi-Naïve Bayesian (SNB) and Random Forest (RF) classifiers trained on medicinal chemists’ selections. The average ROCS score for a 4-fold cross validation of each classifier is reported. **A**: SNB classifier built with medicinal chemistry relevant descriptors (red) is compared to a benchmark Naïve-Bayesian classifier that uses extended connectivity fingerprints and physical chemical properties as descriptors (black). **B**: RF classifier built with medicinal chemistry relevant descriptors (blue) is compared to a benchmark RF classifier that uses extended connectivity fingerprints and physical chemical properties as descriptors (black).

As a first step, we assessed the predictive accuracy of the SNB and RF classifiers compared to benchmark classifiers built with state of the art descriptors that are not as interpretable (Figure 1). For the benchmark classifiers, we trained classifiers with extended connectivity fingerprints (ECFP4) and simple physical properties (ALogP, Molecular_Weight, Num_H_Donors, Num_H_Acceptors, Num_Rotatable_Bonds, and Molecular_FractionalPolarSurfaceArea). The interpretable SNB and RF models compared favorably in predictive accuracy, and in many cases outperformed the corresponding benchmark. The high predictive accuracy of the majority of the classifiers supports the notion that most of the chemists evaluate compounds in an internally consistent manner. For example, for the SNB benchmark, 15/19 models yielded a ROC score >0.7 (Figure 1A, black).

**Figure 2:**
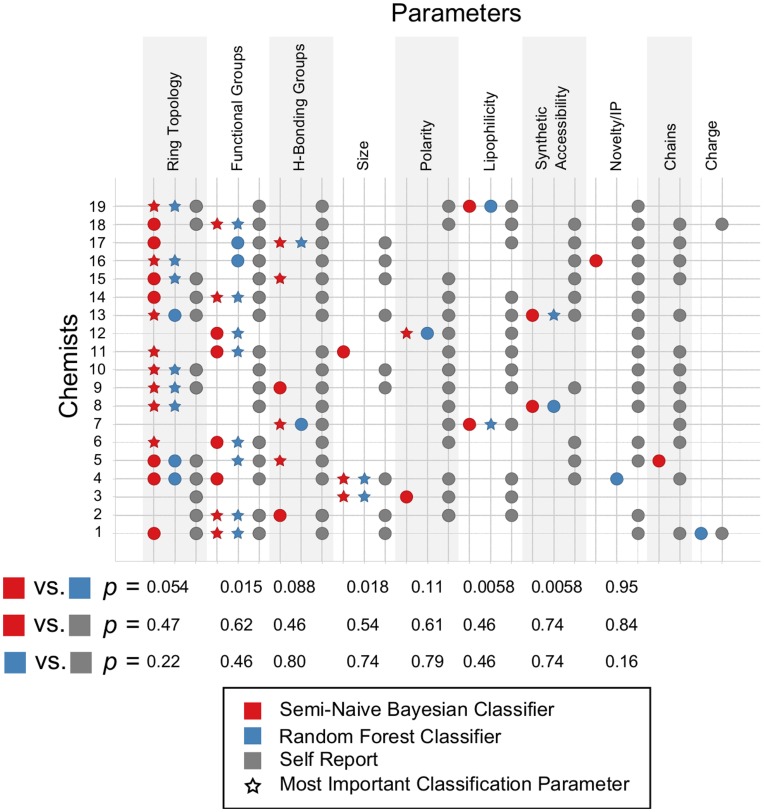
The parameters extracted from the SNB (red) and RF (blue) classifiers are compared with parameters designated as important in chemists’ self-reports (grey). The primary parameters for the classifiers are depicted as stars, and the secondary parameters are depicted as circles. The one-tailed Fisher exact probability test (*p*) is reported for each parameter (except chains and charge), indicating that the SNB and RF parameters show agreement with each other, while the self reported parameters are independent of either of the classifier’s parameters.

The types of parameters used by the SNB and RF classifiers are depicted in Figure 2: we refer to the most important parameter as primary (stars), and all other parameters used as secondary (circles). The descriptors that underlie these parameters are reported in Tables S9 and S10. To our surprise, the majority of the classifiers only used 1–2 types of information. For example, for the SNB classifiers, the majority of classifiers used 2 parameters (16 chemists), while only a few used 1 (1 chemist) or 3 (2 chemists) parameters. The RF classifiers suggest even fewer parameters are important: the majority of classifiers use 1 (9 chemists) or 2 (9 chemists) parameters, while only 1 classifier uses 3 parameters. This suggests that medicinal chemists reduce a complicated problem into a more tractable one by assessing generally just a 1–2 parameters (or types of information) rather than several.

#### Value preferences of SNB models

One of the advantages of our approach is that the SNB classifiers built for each chemist could be visually investigated to bring to light each chemist’s preferences in detail. It should be noted that two models that use the same number of parameters can vary immensely in the complexity or amount of information that they use, although the *type* of information is the same. For example, two chemists might select fragments based on size and polarity. In one case, a complex strategy where interdependencies of these parameters might be used (“large and polar” or “small and nonpolar” compounds are desirable), while another chemist might use a simple strategy where these parameters are considered independently (“large” is desirable, and “highly polar” is desirable). We verified that our SNB classifiers could represent both of these strategies (See Methods and Fig. S2).

**Figure 3:**
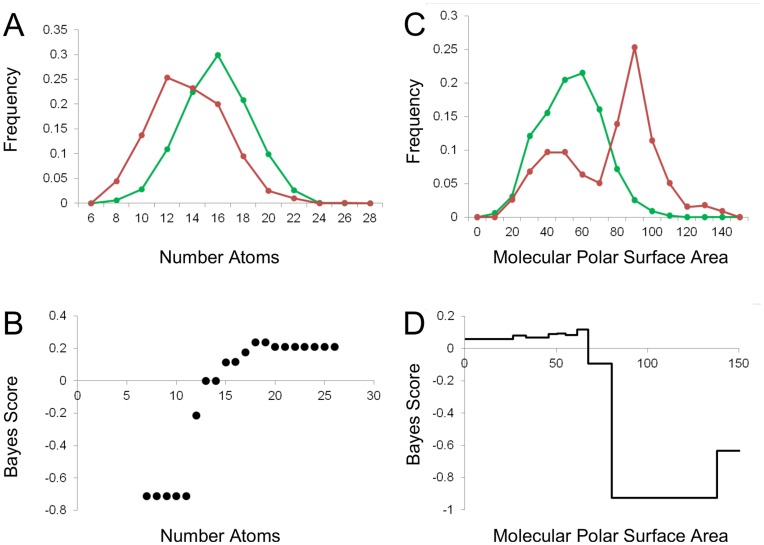
Examples of selection preferences based on simple physicochemical properties, and the corresponding SNB classifiers. **A**: Histogram of number of atoms of fragments selected by chemist 3 as good (green) or bad (red) starting points for drug discovery campaigns. Frequencies are normalized by the total number of selected or unselected compounds, respectively. **B**: Bayesian score versus number of atoms for minimal Bayesian model build for chemist 3. A positive score indicates a favorable number of atoms, while a negative score indicates an unfavorable number of atoms. **C**: Histogram of molecular polar surface area of fragments selected by chemist 12 as good (green) or bad (red) starting points for drug discovery campaigns. Frequencies are normalized by the total number of selected or unselected compounds, respectively. **D**: Bayesian score versus molecular polar surface area bins for SNB classifier built for chemist 12.

We found that in some cases when SNB classifiers were applied to chemists’ decisions, models revealed relatively straightforward preferences. For instance, compounds above a certain cutoff for a particular property are favored, while those below it are disfavored, or vice versa. For chemist 3, size (as measured by the number of atoms) was the most important parameter (Fig. 2); indeed larger fragments were more desirable (Fig. 3A–B). In contrast, modeling revealed polarity to be the primary parameter for chemist 12 (Fig. 2), who showed a strong preference for compounds with a molecular polar surface area less than ∼70 Å (Fig. 3C–D).

**Figure 4:**
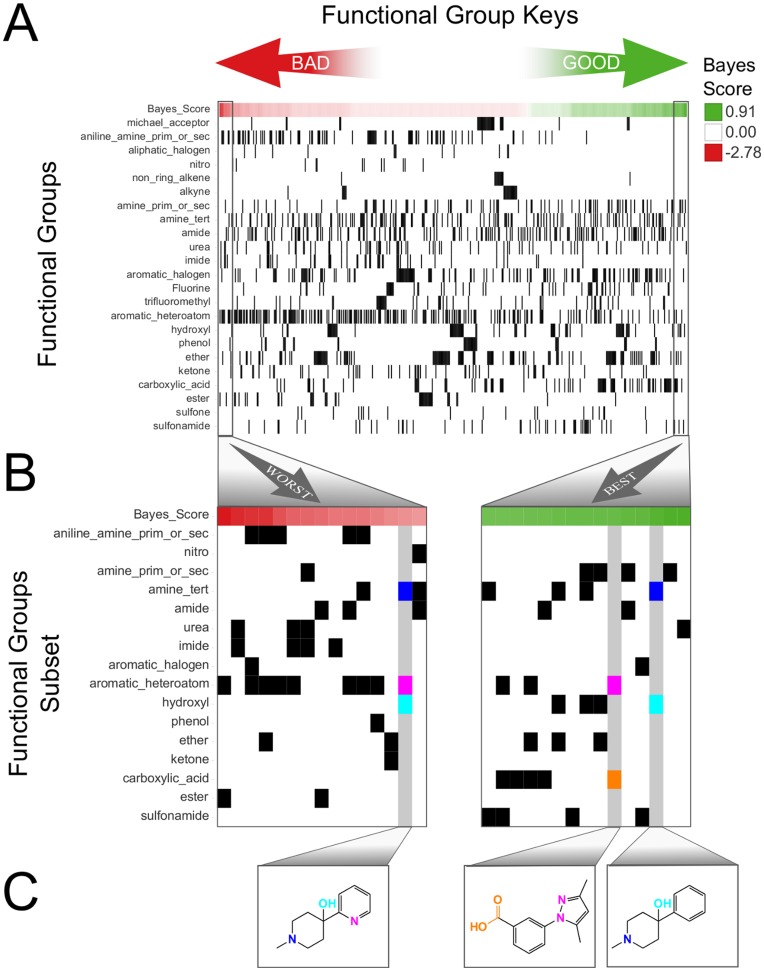
The SNB classifier built using a descriptor subsumed by the functional group parameter is illustrated for chemist 1. Keys that represent the presence (black) or absence (white) of chemical substructures are ordered from negative (bad) on the left to positive (good) values on the right (**A**). The worst and best substructure keys are zoomed in on (**B**). Specific chemical substructures (tertiary amine – blue, aromatic heteroatom – violet, hydroxyl – aqua, and carboxylic acid - orange) are highlighted for one of the worst keys and two of the best keys, and illustrative examples of fragments that would be described by these keys are depicted (**C**).

In contrast to these straightforward preferences, we also observed models that revealed more complex preferences, revealing interdependencies between features. For example, the primary SNB parameter for chemist 1 was identified as functional groups (Fig. 2). Chemist 1’s selections were based on specific combinations of these functional groups (Fig. 4). For example, compounds with hydroxyl groups and tertiary amines were deemed favorable, but if aromatic heteroatoms were also present, they were deemed unfavorable. In fact, chemist 1 in general disfavored compounds containing aromatic heteroatoms. If, however, fragments containing aromatic heteroatoms also contain a carboxylic acid, the compound was seen as favorable. This may be due to the carboxylic acid increasing the attractiveness of the otherwise unfavorable fragment since it might be seen as an especially desirable chemical handle. Importantly, these interdependencies would not have been recognized by our SNB classifiers if the functional groups were considered independently rather than jointly.

**Figure 5:**
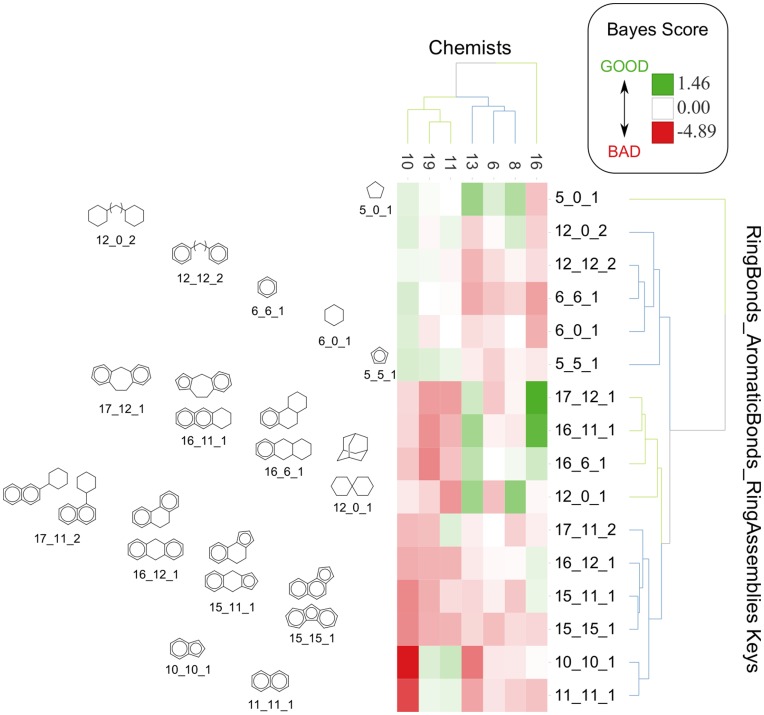
Ring topology SNB classifier comparison between chemists. The most favorable and unfavorable keys for the RingBonds_AromaticBonds_RingAssemblies (RB_AB_RA ) descriptor model, which measures the number of ring bonds (RB), aromatic bonds (AB), and ring assemblies (RA) present in a compound, were examined. Representative scaffolds that correspond to these keys are depicted, and are clustered based on how chemists viewed them. The Bayes score for each models built on individual chemists for each key is reported in a heat map. The favorable keys receive a positive score, while unfavorable keys receive a negative score.

We then investigated how models built with the same parameter compared between chemists. Seven chemists based their decision largely on ring topology; Figure 5 depicts a subset of the most desirable and undesirable values for a descriptor that jointly measures the number of ring bonds, aromatic bonds, and ring assemblies present in a fragment. Representative ring systems that match each descriptor value are depicted. Once again, we see that interdependencies between features are present in ring system preferences. For example, for chemist 19, fused aromatic 6 member rings (11_11_1) are desirable, but when they are connected to an aliphatic 6 member ring (17_11_2), they are undesirable. We note that the rings are grouped together in a chemically intuitive way when they are clustered based on the chemists’ preferences. The chemists were also clustered based on which descriptor values they preferred, revealing the underpinnings of some of the similarities (*S*_MT_) observed between chemists (discussed below). For example, one of the highest similarities observed was between chemist 11 and 19 (*S*_MT_ = 0.47, Fig. S8), and for the subset of values from chemists’ models depicted in Figure 5, they are also the most similar and cluster together first. The ring topology preferences of chemist 10 and 16, on the other hand, are in clear contrast with each other. For example, chemist 10 favors 1–2 ring structures that are not fused, while chemist 16 disfavors these (Fig. 5). Furthermore, chemist 16 highly favors certain fused tricyclic ring structures (17_12_1, 16_11_1, and 16_6_1, Fig. 5) which are disfavored by chemist 10. These differences explain at least in part the low similarity between chemist 10 and 16’s overall selections (*S*_MT_ = 0.19, Fig. S8). Thus, even if chemists use the same parameter to assess compounds, their individual preferences can be quite different. We explore the question of consensus between chemists, which these comparisons foreshadow, in depth in the next section.

In sum, our models show that medicinal chemists appear to have approached a complex decision-making problem regarding the attractiveness of chemical starting points by reducing a massively multidimensional problem space down to one or two salient parameters (or *types* of information). In some cases, these parameters represent a simple pattern of selections, while in others more complex patterns have been identified, such as multiple dimensions being considered jointly.

### Consensus among Chemists

The question of consensus among chemists is a complex one; accordingly we approached it in a number of ways. As a first step, the agreement in parameters used by each chemist during selections was examined. We then investigated the fraction of compounds selected by each chemist. Next, we assessed the similarity of chemist’s selections with themselves (consistency) and with each other (consensus). Finally, we investigated the amount of consensus between chemist selections as a group, and applied the cultural consensus model to assess to what extent individual chemists agreed with the group.

#### Consensus on selection parameters

Because our classifiers revealed which parameters best predicted individual chemists’ responses (Fig. 2), one way in which chemists might show agreement is by relying on the same parameters to guide decisions. For the following analysis, we rely on the SNB classifiers, as their predictive accuracy was on average greater than that of the RF classifiers.

#### One-parameter models

While 14 parameters were available for constructing models, only 9 parameters were actually observed in the SNB classifiers for each chemist; 5 were observed in the one-parameter models. If preference for each parameter is equally likely, we can take .111 (i.e., 1 out of a possible 9 parameters observed) as a hypothetical random probability of a given chemist preferring a given parameter, and compare the observed distribution to this prediction via binomial probability (i.e., compute whether more chemists prefer a particular model than expected by chance). Doing so, we observed that eight chemists’ best one-parameter model utilized ring topology (*p* = .0006). Four chemists utilized functional groups, and another four used hydrogen bond donors/acceptors; these distributions of parameter preferences did not differ from chance levels (*p* = 0.153).

#### Two-parameter models

Similar logic can be used to examine agreement on two-parameter models; here, with 36 unique binary combinations of nine parameters, probability of random agreement is .028. One chemists’ decisions could only be described by a one-parameter model; eleven different two-parameter models were needed to describe the remaining 18 chemists. Of these, more than expected by chance used ring topology plus functional groups (*N* = 5, *p* = 0.0001). Likewise, more chemists used ring topology plus hydrogen bond donors/acceptors than expected by chance (*N* = 4, *p* = 0.001). No other two-parameter model was observed more than expected by chance.

In sum, chemists showed moderate agreement on which parameters were relevant to the decision process.

#### Fraction of compounds selected per chemist

One simple metric of agreement is the fraction of compounds selected by each chemist per batch. The fraction of compounds deemed suitable to carry forward varied widely between chemists, ranging from 7% to 97% (average = 45%), though each chemist was relatively consistent from batch to batch (average standard deviation = 7%, Fig. S6A). This variance between chemists was not related to their ideal library size (Fig. S7A) nor linearly related to the number of targets a chemist had previously worked on (R^2^ = 0.05, Fig. S7B). The fraction passed could, however, be explained by each chemist’s reported selection strategy (Fig. S7C). Chemists who reported selecting only the “best” fragments passed a lower fraction of compounds (0.13±0.07) than chemists that reported excluding only the “worst” fragments (0.61±0.34); those who reported intermediate strategies passed an intermediate fraction of compounds (0.39±0.25).

#### Similarity between chemists’ selections

We next examined how similar individual chemist’s selections were to themselves (consistency) and to each other (consensus) when viewing the same compounds. The modified Tanimoto similarity (*S*_TM_), [38] which ranges from 0 (entirely dissimilar) to 1 (identical), was used to assess the agreement between chemist’s selections. This measure is symmetrical, and therefore equally sensitive to both agreement in selections and rejections. It also takes into account the fraction of selections or rejections for a given comparison; for example, if there is a low number of selections when comparing two chemists, agreement in selections will be weighed more heavily than agreement in rejections. For assessing consistency, a subset of 227 compounds that were present in more than one batch was used. When chemists were compared to themselves, the similarity between selections ranged from 0.37–0.82, with an average of 0.52 (Fig. S8A), indicating moderate consistency. To examine consensus between chemists, the entire set of 3,685 unique compounds was used. When chemists selections were compared to each other, the similarity ranged from 0.05–0.52, with an average similarity of 0.28 (Fig. S8B–D); this indicates substantial disagreement about particular fragments. In sum, chemists were moderately internally consistent in their evaluation of compounds, but the consensus between chemists was low.

#### Consensus in compound selection or rejection

To further investigate these patterns, we calculated the percentage of chemists in agreement on each compound (Fig. S9A). Strikingly, consensus (defined here as 75% of chemists’ agreeing on acceptance or rejection) was reached for only 8% of the compounds reviewed (313 compounds). Moreover, agreement was asymmetrical; 1% of the compounds are considered good while 7% of the compounds are considered bad (Fig. S9A). This is not simply due to a bias in chemists rejecting more compounds than they accept, since on average chemists accepted nearly half (45%) of the compounds. Representative examples of the most undesirable fragments are depicted in Figure S10.

Furthermore, NB models were built on the consensus (≥75% agreement) selections of all chemists (Table S11–12). Separate models were built to identify consensus “good” compounds and consensus “bad.” Models were built with extended connectivity fingerprints (ECFP4). We anticipate that the features identified by consensus selections of chemists for identifying undesirable compounds will be particularly useful in removing undesirable fragments from large collections of compounds, for example, during compound acquisition or when designing focused in-house screens of fragments.

#### Characteristics of high consensus chemists

We then investigated to what extent individual chemists agreed with the group as a whole on compounds where there appeared to be consensus. The cultural consensus model (CCM) is an ideal method for this purpose since it estimates the knowledge - what we term estimated consensus - of respondents on a scale of 0–1 based on the observed agreement between survey answers. [40] (The cultural consensus theory assumes that high consensus is a sign of knowledge (expertise), and thus high-consensus individuals are termed high-knowledge individuals. We use the cultural consensus model as an atheoretical tool to identify members that agree most with the group, so we term them “high estimated consensus” individuals, rather than “high estimated knowledge” individuals.) In this case the survey answers are the fragment selections. As a prerequisite, a single underlying model explaining respondent’s decisions must first be demonstrated. The CCM as implemented in ANTHROPAC 4.0 [41] was used to test for consensus. As expected, a single underlying model did not fit the entire set of selections. By preselecting a set of high agreement compounds (>75% agreement, 313 compounds), a one culture model could be built, as attested by a large ratio of 6.9 between the first and second eigenvalue. In general, an eigenvalue ratio greater than 3 to 1 indicates a single pattern of responses across questions. [42] Importantly, by applying the CCM to the subset of high consensus compounds, an estimated consensus of each chemist was obtained which revealed a vast spectrum of agreement with the group, ranging from 0.07 to 0.66. From this analysis we could also identify a subset of chemists who agreed most with the group; from this subset we could further investigate agreement among high consensus chemists (see below).

**Figure 6:**
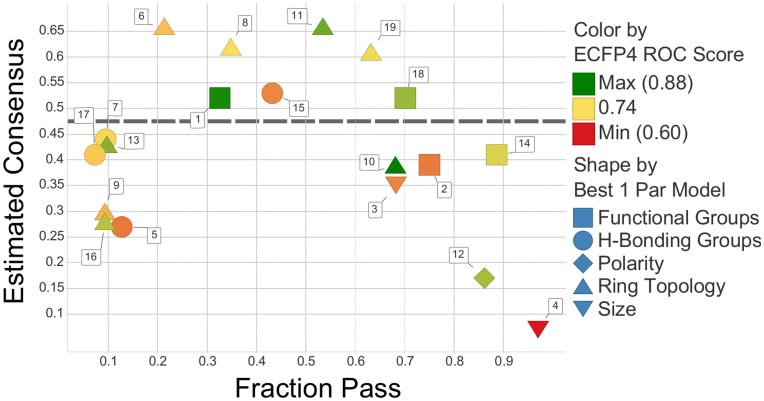
The selection characteristics of chemists with high estimated consensus. The cultural consensus model was applied to a subset of fragments (311) with >75% agreement by chemists. The estimated consensus obtained by this method is plotted against the fraction of fragments passed by chemists for the entire survey. Each shape describes the primary SNB parameter used to reproduce chemists’ selections, and the color depicts the ROC score of naïve Bayesian classifiers built using ECFP4 as a descriptor for each chemist. A subset of high consensus chemists is above the dashed grey line.

We then sought to characterize the selection characteristics of chemists who agreed most with the group. We found that chemists with higher estimated consensus tended to select an intermediate fraction of fragments (∼0.2–0.7, Fig. 6). This is not entirely intuitive, since the majority of compounds that the CCM was built on were rejected compounds, so we might expect a high rejection rate for chemists with high estimated consensus. We might also suspect that chemists with high estimated consensus rely on the same parameters when making selections. Since the ring topology metric was the most common primary SNB parameter for chemists (Fig. 2), it makes sense intuitively that it should be an important property to chemists with the highest estimated consensus. Indeed, ring topology was identified as the primary SNB parameter for the chemists with the highest estimated consensus (chemist 6, 8, 11, and 19), and as a secondary SNB parameter for the chemists with the next highest estimated consensus (chemist 1, 15, and 18). We also noted that a chemist’s estimated consensus was unrelated to the predictability of the chemist’s selections (color-coded, Fig. 6).

We next assessed to what extent the consensus between chemists with high estimated consensus was enhanced compared to the consensus between the same number of chemists selected randomly when considering the entire dataset of selections (Fig. S9B and S9C). The chemists with high estimated consensus (chemist 1, 6, 8, 11, 15, 18, and 19) showed a significantly greater agreement in undesirable compounds (Fig. S9B). The agreement in desirable compounds, however, was no greater than the agreement between chemists selected randomly (Fig. S9C). This reinforces the notion that while there seems to be agreement in what is undesirable, there does not appear to be agreement in what is desirable.

In sum, the overall consensus between chemists is low, and what little agreement there is among chemists seems to be regarding *undesirable* fragments.

### Chemists’ Awareness of Decision Criteria

To assess the extent of chemists’ self-awareness, we compared the parameters reported by chemists to those identified by our SNB and RF classifiers (Fig. 2). The average number of parameters reported by each chemist (8.1±2.2) was much larger than the number of parameters identified by the SNB (2.1 ± 0.5) or RF (1.6±0.6) classifiers for each chemist, which the two-tailed paired sample t-test indicates as significant (*p* = 9.1×10^−10^and *p* = 5.7×10^−10^, respectively). Indeed every single chemist reported properties that were never identified as important by our SNB or RF classifiers. In addition to the properties reported in Figure 2, there were simple parameters (chiral centers and rotatable bonds; included in averages above) and more complex parameters (shape and complexity); not included in the averages above) that were reported by chemists though our approach never identified them as being useful in reproducing selections (Figure S11). Furthermore, Fisher exact probability tests indicated that for each parameter reported in Figure 2, the SNB parameters or RF parameters were independent of the self-reported parameters (*p*-values range from 0.46–0.74 for SNB or 0.22–0.80 for RF, excluding the Novelty/IP parameter, Fig. 2), while indicating that the SNB and RF parameters are consistent with each other (*p*-values range from 0.0058–0.11). In addition, for 12/19 chemists, the primary parameters identified by SNB and RF are in agreement with each other. In other words, there was no systematic relation between the parameters reported by the chemists and those indicated by our modeling, although the parameters identified by the SNB and RF classifiers were consistent with each other.

Perhaps one of the more astounding discrepancies from above, chemist 3 reported that several properties were important, but failed to report that size played any role during selections. Our SNB and RF classifiers both revealed that size, an especially straightforward parameter to assess, was the most important feature in distinguished chemist 3’s selections from rejections (discussed above).

The lack of agreement between the parameters identified as important by SNB or RF classifiers and self-reported parameters for many chemists suggests that medicinal chemists are often unaware of the principal factors that influence their selections.

## Discussion

### Overview

In this paper we explored how medicinal chemists categorized chemical fragments as desirable or undesirable starting points for development into lead compounds. This allowed us to not only investigate the cognitive basis of this important aspect of drug discovery, but also to address basic issues in cognitive science. We focused on three major questions: 1) to what extent, if any, do chemists simplify the problem of identifying promising chemical fragments to move forward in the discovery process? 2) Do chemists agree with each other about the criteria used for such decisions? 3) Can chemists accurately report the criteria they use for such decisions?

### Reducing Complexity

Our results clearly show that chemists greatly reduced the complexity of the problem they were solving. Potentially, one could utilize dozens of parameters (or types of information) to make decisions about fragment suitability. We specifically queried 14 possible parameters in our modeling, 9 of which were used at least once by at least 1 chemist according to either the SNB or RF classifiers. Strikingly, our modeling suggests that the vast majority of chemists only used 1–2 parameters to categorize compounds. In other words, chemists transformed a massively complex categorization problem into a tractable one- or two-dimensional problem. This does not seem to be a bias of our approach since applying our method to simulated classifiers indicated that we could correctly identify at least 4 parameters used in categorization. Furthermore, we used two types of orthogonal classification algorithms to reach these conclusions. It should also be pointed out that SNB models using only 1 parameter can capture rather complex preferences, as in the case of chemist 1’s functional group model. Even so, it is clear that a one parameter model does not use all of the types of information that are available. Category formation based on one dimension, as opposed to many, has been observed in previous psychology experiments as well, even when subjects were asked to use all dimensions when categorizing items [43].

### Consensus among Chemists

We found evidence of moderate agreement among medicinal chemists with respect to the parameters that best modeled their decisions about chemical fragments. For example for the SNB classifiers, eight chemists’ primary parameter was ring topology, and out of 36 possible two-parameter models, two accounted for 47% of chemists. However, we found little agreement with respect to decisions about particular fragments. Only 8% of fragments were accepted or rejected by more than 75% of chemists, the similarity among chemists’ decisions was low, and the cultural consensus model failed to reveal a single underlying model of chemists decisions for the complete fragment set. In other words, even if chemists used the same feature to categorize compounds–which they generally did–they often preferred different values for these features. Moreover, more agreement among chemists was observed regarding what constitutes an undesirable fragment.

We also applied the cultural consensus model to identify individuals that agreed the most with the group as a whole, and to assess the amount of agreement between the chemists. Applying the model to a subset of compounds with high agreement between chemists (≥75%) was necessary in order to obtain a one culture model. It should be noted that the majority of these compounds were deemed undesirable (265/313, Fig. S9A). When we looked at the agreement on desirable and undesirable fragments (for the entire set of survey compounds) between a subset of chemists with high estimated consensus versus a subset of randomly selected chemists, the agreement in the fraction of undesirable compounds was greater, but there was no difference in the fraction of desirable compounds (Fig. S9B–C). These results imply that while there is some agreement regarding undesirable fragments, there does not seem to be a significant amount of agreement regarding desirable fragments. This may be an example of negativity bias – “bad” information tends to be processed longer than “good” information, and stronger memories are formed of “bad” items. [44], [45] Perhaps chemists have retained more knowledge of chemical motifs or properties that literature refers to as undesirable, or that they have had bad personal experiences with, and also paid more attention to these undesirable motifs or properties while they were processing the compounds. In some sense this finding also seems to contradict the notion that chemists tend to recycle privileged scaffolds that they find attractive, ultimately constraining the diversity of chemical series and libraries. [46] It suggests that while individuals have preferences for specific scaffolds, as evidenced by the highly predictive SNB and RF classifiers that were built, these biases are not often shared between chemists.

As mentioned in the introduction, a lack of consensus does not necessarily reflect a lack of expertise, but rather may be a result of the particular problem space under investigation. [24], [26] Three structural factors that contribute to lack of consensus among experts are especially relevant to compound prioritization in drug discovery.

One factor that leads to low consensus is if a single solution does not exist. [24] This is especially true in drug discovery, as evidenced by multiple drugs often being developed for a single target. In light of this, chemists may be playing to their own strengths. In the same way that a master chess player must navigate his chess pieces towards victory, and opens a game in a manner that compliments his own style of play, a medicinal chemist, in the context of a project team, must navigate the path of compounds that he selects to work with towards more optimal properties. The path that one chemist might take likely differs from another, due to the diversity of knowledge and skill sets that an individual brings to the table.

A second factor that leads to low consensus is if the basic science in a field is still evolving. [24] This is particularly true of drug discovery – for example, some topics that have recently garnered much attention that are especially relevant to the current paper are which scaffolds are the most promising in drug discovery, [47] what are the optimal properties of chemical starting points [48] or drug candidates, [49], [50] what are the actual properties of compounds explored by medicinal chemists and how have they varied over time, [51] and how does the subset of chemical reactions that tend to be employed in drug discovery constrain the exploration of chemical space. [52], [53] These studies bear testament that there is still a great deal to learn about the basic science of drug discovery.

A third structural factor that results in low consensus is when experts work in dynamic situations with evolving constraints. [24] In drug discovery, the intended targets of therapeutics are constantly changing, and thus the chemical matter employed to perturb these targets is constantly evolving as well. Furthermore the constraints placed on what defines a suitable therapeutic compound have changed over time. More than ever, researchers are aware of undesirable on or off-target effects, and in many cases are able to interrogate them, ultimately raising the bar for target specificity and minimal toxicity. Indeed, it has been argued that many historically successful therapeutics such as aspirin and acetaminophen would not be considered suitable therapeutics in the current drug discovery environment [54].

### Tying Complexity Reduction and Consensus Together: Goal Derived Categories

One interesting way to frame both the complexity reduction and consensus results is in terms of goal-derived categories. Goal-derived categories unite otherwise diverse entities in the service of a particular goal; for instance, shirts, novels, and toothbrushes are all *things to pack in a suitcase*. [55] Like common taxonomic categories (e.g., *dog, tree, car*), goal-derived categories have been shown to exhibit prototype structure (i.e., some exemplars are more prototypical or “better” members of the category than others). However, different factors determine prototype structure for the two types of categories. The best examples of taxonomic categories tend to be similar to many other members; they represent the central tendency of the category. In contrast, the best examples of goal-derived categories tend to be instances that satisfy specific ideals–i.e., instances that have characteristics that serve the goal optimally. Another determinant of typicality for goal-derived categories is frequency of instantiation, or how often an instance is encountered as a member of the category.

It’s plausible that our chemists are deciding whether or not the target fragments are members of the goal-derived category *promising fragments for drug discovery follow-up*. If so, chemists should make decisions based on how well fragments satisfy ideals, and their frequency of instantiation as promising leads. [56] In our case, ideals are characteristics that fragments should possess if they are considered desirable for lead development (e.g., synthetic accessibility, facile derivatization, etc.), whereas the frequency of instantiation could be thought of as the number of times a chemist encounters a compound or chemical motif and associates it with being desirable or undesirable for lead development. Our results show that although chemists tend to converge on a small subset of possible parameters for making these decisions, they show little agreement on the optimal values for these parameters. This lack of consensus could arise from several sources.

First, the complexity of what constitutes an attractive starting compound for optimization in the drug-discovery process may have led to differences in the ideals that chemists sought to optimize. Second, people often optimize more than one ideal during categorization, [55] and it is likely that in our case individual chemists may also weight the importance of multiple ideals differently. For example, one chemist might place more emphasis on making sure a fragment can be easily evolved, while another might place more emphasis on reducing potential toxicity. Furthermore, chemists may also associate different parameters with these ideals. For instance, two chemists may both desire a fragment that specifically interacts with a target, and one chemist may view shape as an important feature, while another may view hydrogen bonding interactions as more important.

One reason that chemists might share the same ideals (e.g., synthetic ease), while favoring different values for these ideals may be due to their personal experience (e.g., synthetic transformations they are most familiar with). In other words, the distribution of frequencies of instantiation is undoubtedly different for individuals, and this may be reflected by different optimal values. If chemists have worked in different target areas, they may have been exposed to different chemotypes or functional groups. [47], [57] A follow-up questionnaire was employed to identify which target areas survey takers had experience in (Fig. S12). The diversity of backgrounds that was observed may have lead chemists to view different motifs that are commonly encountered while working on specific drug target areas as “druglike,” privileged, or easy to work with. It is also likely that even if chemists have been exposed to the same target classes during their professional careers, they may extract different features from desirable compounds during learning based on their backgrounds [58], [59].

There is likely a complex relationship between a chemist’s ideals and the parameters that were identified by the SNB and RF classifiers as indicative of their selections. In specific cases, however, by visually inspecting the individual SNB classifiers, it is tempting to extrapolate ideals for individual chemists based on the ideal’s impression upon optimal values for specific parameters. For example, in one model (chemist 12), compounds with a polar surface below a certain threshold are desirable, and those above it are undesirable. This ideal has been stated in drug design literature: the polar surface area of a drug-like compound should not be too high, as it negatively impacts oral bioavailability [60], [61].

### Chemists’ Awareness of Decision Criteria

Chemists were largely unaware of the factors that influenced their decisions about compounds. Chemists reported that they relied on more parameters than they actually did, according to the SNB and RF classifiers, and there was little agreement overall between the properties chemists identified and the parameters that predicted their decisions. We should point out that for specific instances parts of the self reports were extremely accurate. For example, chemist 10 disclosed a list of features largely related to the ring topology parameter. This list was written down before evaluating the first set of compounds, and was used as a reminder throughout the exercise. Although the reported features were evident in chemist 10’s selections, several other self-reported parameters were not identified as important. In stark contrast to chemist 10 is a chemist who reported that sometimes, in addition to the specific properties they reported, they trusted their “gut feeling.” Perhaps, since a predictive model could be built for this chemist, this “gut feeling” is really based on previous unconscious learning. As discussed in the introduction, such lack of awareness of the factors affecting decisions is fairly characteristic of human decision-making in complex situations. Furthermore, experts have also been described as inarticulate about the process used to make decisions. [62] In our study, the intuition was clearly rooted in expertise: a compound is unlikely to “strike” anyone as promising or unpromising unless one has extensive record of performing such complex evaluations. This raises an interesting question: would novice chemists be more or less aware of the parameters they based their decisions on than experts proved to be? If lack of expertise makes the compound evaluation a slower, more effortful process, we can expect novices to be more accurate in reporting the parameters that influenced their decisions - unless they are put under time pressure forcing them to rely on their fast (non-expert) intuitive thinking. Another question is why the participants overestimated the number of parameters they relied upon. Perhaps, if the self-reports were based on post hoc rationalization of already made decisions, the reports were driven by a meta-expectation about the average number of parameters an expert *should* consider in such a situation in order to arrive to a justified decision. If chemists reading this paper find themselves surprised at the small number of parameters their colleagues used, their reaction informally testifies to the existence of that very meta-expectation.

### Implications and Conclusions

We found that chemists tend to exhibit stable decision bias by consistently considering one or two parameters rather than many. What does this imply for drug discovery? As discussed by Gigerenzer & Brighton, [8] stable bias is sometimes preferable over optimization strategies. Both stable bias and over-fitting the data with an excessive number of parameters contribute to the overall amount of predictive error. A simple strategy that avoids over-fitting by accepting bias can in the end turn out to be more successful. This principle lays ground for many “less-is-more” effects, where ignoring parts of available information leads to a more accurate prediction. As Hertwig & Herzog put it, “the art is to ignore the right information.” [63] What should and shouldn’t be ignored is determined by the specific problem one is trying to solve. Under the approach of ecological rationality, [8], [64] simple and complex decision strategies should be compared not in terms of overall adherence to domain-general principles of logic, but based on how well they fare in specific environments. This leads one to question whether drug discovery is a good domain for the simplified decision strategies that chemists are using. Future studies aiming to address this might entail associating some measure of success with compounds, and comparing the ability of chemists versus potentially more complex computational protocols in selecting desirable chemical starting points.

As discussed earlier, drug discovery is a multiple solution problem space, and individual chemists can use their unique strengths to explore chemical space while optimizing lead compounds. That being said, a problem arises when a personal bias does not lead one down a fruitful path. Consequently, our research has implications for the education of medicinal chemists and the structure of project teams. When hiring young chemists to practice medicinal chemistry, pharmaceutical companies tend to prefer a strong organic synthesis background over all other skill sets, even over a medicinal chemistry background. [65]–[68] It is thought that skills perceived as secondary can be taught on-site, post-employment. [65]–[68] Thus, it may be beneficial to expose medicinal chemists to diverse chemical motifs, and how they have been advanced in the industry, in order to broaden the toolbox of interesting chemical starting points for individual medicinal chemists. Furthermore, project teams should be aware that if one chemist’s influence is dominating how chemical space is explored, the chemist’s personal bias may not necessary lead down a beneficial path, although that path may exist. As such, it may be advantageous to rely on two to three chemists with different backgrounds and synthetic strengths in identifying interesting series of initial compounds to explore, and then ultimately pursuing the most promising leads once additional knowledge has been generated.

The chemical space available for exploration by medicinal chemists in the search for therapeutics is vast. This search process serves as a real-life example of humans making decisions about the unknown, based on limited knowledge, which holds huge potential for reward. Inherent in this search is the reduction of complexity to a manageable number of dimensions. Here we have revealed in part how experts have cognitively tackled this daunting problem, and identified in detail the parameters employed when prioritizing which compounds to explore during drug discovery. By focusing on how humans explore, interact, and understand chemical space, rather than solely viewing drug discovery as a sterile process where the “right” answer or compound will eventually emerge, it is hoped that the human biases inherent in drug discovery may be leveraged or mitigated to the advantage of the discovery of therapeutically beneficial chemical matter.

## Methods

### Overview

We sought to illuminate which molecular features influence the attractiveness of a compound to a chemist by statistically interrogating the choices made by individual chemists asked to review ∼4,000 chemical fragments (compounds<300 MW), and select fragments they would be willing to carry forward in a lead discovery effort. Fragments are ideal for this purpose as they are less complex than larger compounds, with fewer potentially conflicting features, allowing easier interpretation of chemists’ decisions. Furthermore, a survey of a given number of fragments covers a much greater fraction of the possible fragment chemical space than a survey of the same number of small molecules would cover of possible small molecule chemical space, suggesting that models derived from the study may be more transferable. A study based on fragment selections is also especially relevant to the pharmaceutical industry as many companies now use fragment based screening as a method to identify interesting chemical scaffolds, [69]–[71] and the number of hits is often high enough to warrant prioritization of a subset of fragment hits. [72] We simplified the selection exercise by not including biochemical information that might influence selections. In our case, the selections should solely rely on the structures of the fragments that are presented, and how chemists assess whether they would want to explore derivatives of such fragments. Here we describe the surveys, follow-up questionnaires, SNB and RF classifiers, and validation of the classifiers.

### Fragment Set Preparation

Fragment-sized compounds (MW≤300) were selected from the Novartis archive and filtered based on physicochemical property cutoffs (ClogP, number of hydrogen bonding groups, etc.) and undesirable substructures based on in-house and external knowledge (e.g., epoxides). In addition, the number of chemical handles, diversity, chemical attractiveness (based on in-house Bayesian models trained on medicinal chemists assessing HTS hit compounds) were used to select the compounds. The fragments were further required to have prototypes in the archive. The identity, purity and solubility of the compounds were determined by NMR, and additional profiling included binding to a CM5 BiaCore chip. The results from the BiaCore and NMR experiments were used to filter for acceptable compound quality control (QC) and solubility, respectively, yielding a set of ∼3,700 compounds for further analysis and selection by chemists.

### Survey

The ∼3,700 molecules above were separated into eight batches. Previous experience with interactive selection of attractive fragments by chemists suggested 500 molecules was an optimal batch size for visual evaluation. 227 molecules were sent more than once (in different batches), in order to assess consistency in chemists’ selections when they viewed the same compound on separate occasions.

The molecule batches were created in the order BiaCore and NMR profiling proceeded and imported into ICM sessions (internally modified version of ICM Chemist from MolSoft [73]). ICM offered a chemically aware spreadsheet that could be toggled into an interactive structure grid where cells could be selected and table position navigated with keyboard (in addition to mouse) to minimize fatigue. The grid could be interactively resized to show the desired number and size of molecules on different displays. By default, upon opening a session, the view was in grid mode, with compound structure, ClogP and number of heavy atoms displayed (Fig. S1). All molecules were deselected by default. Before starting, each user was asked to shuffle the molecules into a new random order via a hyperlink in the session, to reduce order bias (first molecules receiving more attention than last) in the user group as a whole. To select a molecule, users needed to press the number 1 key and to undo selection, 0. The session could be saved and work continued at another time. Upon completion, the user was asked to upload the session to a shared location.

Chemists were invited to participate in the selection panel via an e-mail message from senior chemistry management. 19 chemists evaluated at least 7 out of the 8 batches of compounds. They were located at 3 Novartis sites: Basel (Switzerland), Cambridge (MA, USA), and Emeryville (CA, USA). They were all of doctorate-level training, and had various levels of experience working in industry. The target areas that the chemists had worked on are reported in Figure S12. The molecule batches were sent to the panel of chemists over two months. Participants were asked to pick molecules they would be willing to follow up if they were hits in a fragment screening campaign. Participants were purposely given vague instructions on how they might assess each fragment, suggesting they might consider things like whether fragments were sufficiently functionalized so that they could interact with binding sites, whether they could be grown, and their shape. No guidance was given about number of molecules to select. Selections from the uploaded ICM sessions were extracted with an ICM script into ASCII files and further processed with Pipeline Pilot 8.0 [74].

### Follow-up Questionnaire

After completing the fragment surveys, chemists were asked to complete a web-based follow-up questionnaire that consisted of both open-ended and closed-ended questions. A number of items on the questionnaire were based on preliminary findings from our classification models, although we did not share any of our results with the participating chemists.

### Simulated Classifiers

It has been demonstrated that great care must be taken when attributing meaning to features used by classification algorithms. [75] Thus, simulated classifiers with known selection preferences were built to validate that classification models would be able to correctly extract what parameters were used during compound selection, prior to deriving classification models based on each of the chemists’ selections. The simulated classifiers categorize fragments as desirable or undesirable fragments, and those category labels are then used to build classification models (SNB or RF) that would hopefully recapitulate the criteria used to build the fragment sets. The simulated classifiers assessed the same fragment set as the chemists, and selected desirable and undesirable compounds based on predefined criteria. For each compound, the classifier first assessed whether the compound fell into the desired chemical space (i.e., passing specific physical chemical property cutoffs, not possessing undesirable substructures, etc.), and then classified the compound as good or bad. To build noise into the classification to more realistically represent human decisions, desirable and undesirable compounds were misclassified 5% of the time.

The first set of simulated classifiers selected compounds based on 1–4 parameters (Table S1). The purpose of these classifiers was to assess how accurately SNB and RF classifiers could identify the type and number of parameters being used by the simulated classifier.

A second set of simulated classifiers was designed in order to assess the ability of SNB classifiers to correctly classify compounds when there are interdependencies between attributes. The simulated classifiers selected fragments as good or bad based on 1–2 attributes, with selection patterns varying from simple to complex. The two attributes of the compounds that were used by the simulated classifiers were number of atoms (size parameter) and molecular polar surface area (MPSA, polarity parameter). Four selection criteria for desirable fragments were assessed (depicted from left to right in Fig. S2A) using the following pseudocode:

1. number of atoms≥15
2. MPSA<60
3. number of atoms≥15 AND MPSA<60
4. (number of atoms≥15 AND MPSA≥60) OR (number of atoms<15 AND MPSA<60)

The last selection strategy is an example of the classical XOR (exclusive ‘or’) nonlinear problem [76].

A third set of simulated classifiers which selected fragments as good or bad randomly was used to ensure that the SNB or RF classifiers identified legitimate parameters used during selections. We tested three different cutoffs for the random classifiers to use for the fraction of fragments to select (0.1, 0.5, 0.9).

### Classification Models

Pipeline Pilot 8.0 [74] was used to build all classification models based on either simulated classifier or chemists’ selections. A 4-fold cross validation was carried out for all classifiers as follows. The survey responses were divided into 4 training and test sets (Table S2), and after training a model, the average area under the receiver operating characteristic curve (ROC score) for the test sets was used to assess a given model’s predictability (for example, Fig. 1).

#### Descriptors and parameters

72 medicinal chemistry-relevant descriptors (Table S3) were assessed or developed in order to more readily elucidate what properties (e.g., number of chemical handles, ring topology, number of hydrogen bond donors or acceptors, etc) needed to be included as descriptors in order to build accurate classification models for each individual chemist. Many of these descriptors were directly calculated with standard components available in Pipeline Pilot 8.0. A number of these descriptors, however, were either obtained by combining values calculated by Pipeline Pilot into a fingerprint, so that they were considered jointly, calculated by a stand alone program, or calculated with an in-house Pipeline Pilot protocol. Some of the less straightforward descriptors are described in Tables S4 (chemical handles) and S5 (functional groups).

For semi-naïve Bayesian (SNB) classifiers, it was necessary to consider a number of descriptors jointly by combining individual values into a fingerprint (for example ring bonds, aromatic bonds, and ring assemblies: RB_AB_RA, illustrated in Fig. 5), in order to model interdependencies. This is not necessary for random forest (RF) classifiers, since interdependencies are encoded in the structure and splits of each tree. Thus, while RF classifiers used the same descriptors, they only needed to be used independently when training the RF. Continuous descriptors were binned into ∼5 bins prior to training the RF classifier.

In order to identify what type of information was used to classify compounds, each descriptor is mapped to one or more general parameters. For example, both molecular weight and number of atoms map to the parameter “size.” In this way, descriptors identified as important by a classification model can then be converted to parameters that they relate to, elucidating the type of information used during classification by a medicinal chemist. A total of 14 parameter classes were defined, namely ring topology, functional groups, h-bonding groups, size, polarity, lipophilicity, synthetic accessibility, novelty/IP, chains, charge, chiral centers, complexity, rotatable bonds, and shape.

For the accuracy benchmark models for both SNB and RF, extended connectivity fingerprints with diameter 4 (ECFP4) were used in combination with simple physical properties (ALogP, Molecular_Weight, Num_H_Donors, Num_H_Acceptors, Num_Rotatable_Bonds, and Molecular_FractionalPolarSurfaceArea) as descriptors to train a naïve Bayesian (NB) or RF classifier, respectively. The ECFP descriptor takes into account all substructures of a compound, and has been well established as input to classification models in accurately separating classes of compounds. [77] While the ECFP descriptor lends itself to accurate model construction, the resulting models are not readily interpretable in terms of what general parameters might be important. Thus, classifiers constructed with ECFPs stand as excellent accuracy benchmarks that other more interpretable models might achieve.

#### Semi-naïve Bayesian (SNB) classifiers

SNB classifiers were developed in order to generate models that are easily interpretable like their progenitor, naïve Bayesian models, but also capture interdependencies of attributes that naïve Bayesian models cannot. [76] Our classifiers are semi-naïve in the sense that features are often considered jointly rather than independently, and we perform a feature subset selection on the descriptors that are used by the classifiers in order to remove redundant descriptors that will lower overall model accuracy, [78] and to remove features that do not contribute to selections.

In all, 192 classifiers were first built for each chemist using one or more medicinal chemistry relevant descriptors. It would not be feasible to test all descriptors in all possible combinations, so a number of avenues were used for focusing on the most relevant models to build. In some cases all combinations of a few uncorrelated descriptors were considered. In addition, a number of classifiers were designed by combining descriptors that 1) showed some enrichment in desirable or undesirable fragments for at least one chemist and 2) were not correlated with each other. The enrichment of a particular descriptor could readily be assessed by the magnitude of a ROCS score for a model based on that descriptor; all descriptors that resulted in ROCS scores >0.6 for at least one chemist were tested in combination with other descriptors. To assess for correlation between descriptors, we used a PCA analysis of the descriptors, various correlation statistics, and expert knowledge. Thus, two descriptors that are known to measure similar properties (say number of atoms, and molecular weight), were not paired together. This was not done in a rigorous way, however, because even if two descriptors that are somewhat related to each other are paired together, if the information that they provide is redundant rather than complementary, then the resulting model’s predictive accuracy will likely be the same or lower than that of a model that uses only one of the said descriptors, and the model with redundant descriptors would not be selected during feature subset selection (see below).

In order to identify the most important parameters for each chemist, we developed a feature subset selection method that identifies the SNB classifier that only uses essential descriptors (Fig. S3). As mentioned before, each descriptor is mapped to a more general parameter. Thus each model can also be thought of as built from one or more parameters. In the first step of selection, the best 1 parameter model is selected (N = 1) from all possible 1 parameter models, as assessed by the average ROCS score from the 4-fold cross validation of each classifier. It is then compared to the best 2 parameter model (N+1). If the best 2 parameter model is significantly more accurate, as indicated by the ROC score increasing by >0.009, then N is incremented (N = 2), and the current N parameter model is compared to the N+1 parameter model. A cutoff of 0.009 was used as it resulted in the selection of SNB classifiers with parameters that were known to be important for the simulated classifiers, while not selecting SNB classifiers that contained parameters that did not relate to the simulated classifier. This process is continued until the predictive accuracy of the N+1 parameter model does not increase more than 0.009. The parameter identified for the N = 1 SNB classifier is termed the primary parameter. All other parameters (if any) are termed secondary.

We also note that we took into account the possibility of local minimum when selecting features to include in the SNB classifier. For example, a local minimum might be found if a 2 parameter model is not significantly more accurate than a 1 parameter model, but a 3 parameter model is. In order to avoid local minima, the accuracies of all models were computed regardless of the number of parameters in the model for each chemist, and the accuracy of the selected SNB classifier was compared to that of the most accurate classifier. In most cases, when a local minimum was obtained, SNB models with an intermediate number of parameters were missing, and these models were added to the analysis.

Prior to applying this method to chemists’ selections, it was first validated on simulated classifiers, which separated compounds based on known parameter preferences. The first set of classifiers tested whether the classifiers could identify the correct type of information being used by the simulated classifier, and what number of parameters classifiers could identify. The predictive accuracy of the classifiers trained with the medicinal chemistry relevant descriptors compared well with benchmark classifiers (Fig. S4) trained with ECFP4 descriptors and simple physical properties (ALogP, Molecular_Weight, Num_H_Donors, Num_H_Acceptors, Num_Rotatable_Bonds, and Molecular_FractionalPolarSurfaceArea). The number and types of parameters identified as important by the SNB classifiers (Fig. S5) were in good agreement with the criteria used by each of the simulated classifiers to select compounds (Table S1). The descriptors that underlie the parameters are reported in Table S7. This study demonstrated that our method could correctly identify up to 4 parameters (or types of information) used to separate compounds. As we show below, this was more than enough to recapitulate the chemists’ selections.

We used a second set of simulated classifiers to assess the ability of the SNB classifiers to correctly classify compounds when interdependencies were present between attributes. Four different SNB classifiers were trained on the simulated classifiers’ selections. Two of the SNB classifiers assessed consisted of one attribute (Atoms Fig. S2B, or MPSA Fig. S2C). Another SNB classifier included both Atoms and MPSA (Fig. S2D). A final SNB classifier, considered Atoms and MPSA jointly (Fig. S2E). For each of the simulated selection strategies, the SNB classifier that would be selected by our feature subset selection method is boxed (Fig. S2). For the simple selection strategies based solely on one attribute (Atoms or MPSA), the classifier trained using only that attribute is selected. In the third scenario, where fragments with ≥ 15 atoms and MPSA<60 are considered desirable, the classifier that uses both Atoms and MPSA (independently) is selected. In the fourth scenario, the XOR case, the classifier that considers both number of atoms and MPSA jointly is selected.

This study reveals that when attributes are considered jointly, SNB classifiers can recapitulate complex patterns that might result from dependencies between attributes. Indeed, these types of patterns are investigated in the Results section for other attributes that were considered jointly, and turned out to be important in chemists’ selections (see “Value Preferences of SNB Models”, as well as Figures 4 and 5).

A third set of simulated classifiers tested how SNB classifiers behaved when fragments were selected randomly. When SNB classifiers were applied to the random simulated selections, no ROC score was obtained that was greater than 0.55 (Table S6). This sets a threshold for ROC scores that we can consider better than random. Indeed, all of the models built on the chemists selections were higher in accuracy, suggesting that our method is indeed robust, and that ROC scores>0.55 will only be obtained when selections are not randomly made.

Bayesian models have been discussed in detail elsewhere, so we will only highlight important equations for our work. The Bayesian Score for a given feature is:
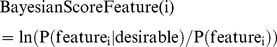
(1)and the total Bayesian score over all features is:



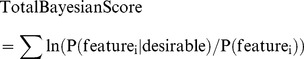
(2)In our case, the Bayesian score for a feature is positive if a feature or bin is desirable and negative if it is undesirable. When a compound is being classified by a SNB classifier, if the total Bayesian score is positive than it is scored as desirable, and if it is negative it is scored as undesirable. The Bayesian scores for specific features or bins in SNB models were useful in interpreting and visualizing specific models (Fig. 3, 4, 5).

#### Random forest (RF) classifiers

In order to independently validate the results from the SNB classifiers, we employed RF classifiers as an orthogonal classification method. The Learn RP Forest model component was used in Pipeline Pilot 8.0 [74] to generate the RF classifiers. The descriptors used were the medicinal chemistry relevant descriptors (mentioned above), except continuous descriptors were binned into 5 bins, and joint descriptors were not used (since dependencies can be encoded by the tree structure and splitting patterns). The model used is termed a balanced forest of random trees. [39,80] For each tree, a minimum of 10 samples were allowed per node, the maximum tree depth was 20, the Gini index was used to choose the split for each node, [7] and the weighting method was uniform. In each Forest, there were 500 trees, bagging was used, [3] the class sizes were equalized, [9] and the number of descriptor properties to consider for use as a split criterion within each tree was set to the square root of the total number of descriptors. [9] Three trials (with 3 random seeds) were used for each of the 4 sets of training and test sets.

For the RF classifiers, the percent selection frequency of each descriptor was used as a measure of that descriptor’s importance. This was averaged over the 3 trials for each of the 4 training sets, and the average percent selection frequency was converted to a z-score for each model. A cutoff was then determined to ascertain which descriptors were important. This cutoff was established by using simulated classifiers which selected compounds based on known parameter preferences, and then observing at which value the parameters of importance lied above the cutoff, and parameters not used by the simulated classifier lied below the cutoff. We found that a cutoff of 2.1 worked well to separate important descriptors from unimportant descriptors for the simulated classifiers (see below), and subsequently used this cutoff to identify important descriptors for RF classifiers trained on the chemists’ selections as well. The parameter corresponding to the descriptor with the highest z-score is termed the primary RF parameter, and all other parameters above the 2.1 cutoff (if any) are termed secondary.

In all cases, the parameters that relate to the types of criteria used by the simulated classifiers to categorize fragments as desirable or undesirable were identified. In some cases, however, unlike the SNB classifiers, additional parameters were deemed important as well. The selection of these parameters could be rationalized, however, when the descriptors underlying these parameters were investigated. For example, in the case of the Molecular_PolarSurfaceArea and Atoms_MPSA simulated classifiers, the Functional Groups parameter was incorrectly identified by the RF classifier. The descriptor that mapped to the Functional Groups parameter in this case was the sulfonamide descriptor (Table S8, which counts the number of sulfonamides present). Although sulfonamides were not specifically selected by the simulated classifiers, their presence correlates somewhat with polar surface area (the more sulfonamides, the greater the polar surface area), so their selection makes some sense. Similarly, for the Substruct_FG simulated classifier, the Ring Topology classifier was incorrectly identified as important. The descriptor that was used in this case was Num_AromaticRings (Table S8, which counts the number of aromatic rings present). This makes sense because the simulated classifier deemed aromatic amines in 5-membered rings as undesirable, so the number of aromatic rings present will be roughly related to this. In summary, while the RF classifiers identify the correct parameters, they also sometimes identify additional parameters due to descriptors that correlate somewhat with properties that were used during selections. This was not observed with the SNB classifiers.

## Supporting Information

Figure S1 **Simulated fragment selection session.**
(BMP)

Figure S2 **A: Simulated classifiers selected fragments as good (green) or bad (red) based on thresholds for molecular polar surface area (MPSA) or number of atoms.** The Bayes score of different bins for Naïve Bayesian models built using atoms (**B**), molecular polar surface area (**C**), atoms and molecular polar surface area independently (**D**), or atoms and molecular polar surface area jointly (**E**) are depicted. For the exclusive or (XOR) case (fourth panel in all rows), only the semi-naïve Bayesian model can correctly represent the simulated classifiers pattern. The ROCS score for each of the models is reported in corresponding panel for that model. The panel of the classification model that would be selected by the feature subset selection method that was employed is boxed with a black square.
(PDF)

Figure S3 **Feature subset selection for SNB classifiers.** N is set to 1, and the best N parameter model is selected. It is then compared to the best N+1 parameter model. If the ROC score of the best N+1 parameter model is significantly more accurate than the current best N parameter model (difference>0.009), then N is incremented, and the process is repeated. If not (difference<0.009), then the current best N parameter model is selected.
(PDF)

Figure S4 **Predictive accuracies for SNB and RF classifiers when trained on selections made by simulated classifiers.**
(PDF)

Figure S5 **The parameters extracted from the SNB (red) and RF (blue) classifiers for selections made by simulated classifiers.** The primary parameters for the classifiers are depicted as stars, and the secondary parameters are depicted as circles.
(PDF)

Figure S6 **The fraction of compounds selected as desirable by each chemist.** **A**: The fraction of compounds selected per batch by each chemist. The average fraction pass is 0.45 and the average standard deviation is 0.07. **B**: Histogram of the number of chemists that passed a specified fraction of fragments per batch.
(PNG)

Figure S7 **Relating the fraction of compounds selected as desirable to various factors.** **A**: The average fraction of compounds passed per batch for chemists with different ideal fragment library sizes. **B**: The fraction of compounds passed versus the number of targets a chemist had worked on. **C**: The average fraction of compounds passed per batch for chemists with different selection strategies. Self-reports were used to obtain the ideal fragment size, number of past targets, and selection strategies.
(PNG)

Figure S8 **The similarity of selections when comparing chemists’ selections to themselves and to each other.** A histogram of the modified Tanimoto similarities (*S*_MT_) comparing chemists to themselves (**A**). Similarities between chemists depicted as a heat map (**B**) and in table form (**C**). A histogram of modified Tanimoto similarities obtained between chemists (**D**). Two clusters formed by chemists using a modified Tanimoto similarity cutoff of ≥0.44 (**E**).
(PNG)

Figure S9 **A comparison of consensus in desirable or undesirable fragments.** **A**: The fraction of consensus good (green) or bad (red) compounds that pass when a given threshold for consensus is used. At all thresholds, there are more consensus good than consensus bad compounds. **B**: The fraction of consensus bad compounds for seven chemists with high estimated knowledge (red) versus seven randomly selected chemists (black) **C**: The fraction of consensus good compounds for seven chemists with high estimated knowledge (green) versus seven randomly selected chemists (black).
(PNG)

Figure S10 **A selection of the fragments deemed worst by the group.** The number of yes and no votes is below each structure.
(TIF)

Figure S11 **Parameters that were included in self-reports but not identified as important by SNB or RF models for each chemist.** Note, “Diversity” and “Metabolic Stability” were self-reported, but attempts were not made to model these parameters.
(PDF)

Figure S12 **The types of targets chemists have previously worked on, as self-reported in the follow-up questionnaire.**
(TIF)

Table S1 **Simulated classifiers with 1–4 rules for identifying good fragments are listed.**
(DOC)

Table S2 **Training and Test sets for 4-fold cross validation.** The eight batches of compounds that were surveyed were jackknifed as follows to yield 4 training and test sets.
(DOC)

Table S3 **Descriptors (72) used for building minimal Bayesian models.** The parameter(s) that the descriptor is subsumed by is reported, as well as whether it was calculated using Pipeline Pilot (PP) or RDKit. Some descriptors were derived from combining or mathematically manipulating metrics previously calculated by Pipeline Pilot or RDKit (Custom).
(DOC)

Table S4 **Chemical handles.** For the chemical_handles descriptor, chemical handles that a chemist might manipulate were counted. Specific types of substructures were only considered chemical handles if they were located on the core, on an R-group, or both.
(DOC)

Table S5 **Functional groups included in functional group key.** A fingerprint of medicinal chemistry relevant functional groups (smarts_fp) was developed to characterize the functional groups present or absent in a compound. SMARTS substructures were used to identify the presence of substructures, and these were combined into a functional group key. If the functional group is present in the fragment, the value for it in the key is 1, while if it is absent the value is 0. This is the descriptor used in the model illustrated for chemist 1 in Figure 4.
(DOC)

Table S6 **ROC Scores obtained for random simulated classifiers that passed different fractions of compounds.**
(DOC)

Table S7 **Descriptors identified as important by the SNB classifiers for selections made by the simulated classifiers.** The best 1 parameter model is designated 1_paramater (this corresponds to the descriptor that underlies the primary parameter), and the final SNB model is designated N_parameters.
(XLSX)

Table S8 **Descriptors identified as important by the RF classifiers for selections made by the simulated classifiers.**
(XLSX)

Table S9 **Descriptors identified as important by the SNB classifiers for selections made by chemists.** The best 1 parameter model is designated 1_paramater (this corresponds to the descriptor that underlies the primary parameter), and the final SNB model is designated N_parameters.
(XLSX)

Table S10 **Descriptors identified as important by the RF classifiers for selections made by the chemists.**
(XLSX)

Table S11 **ECFP4 features extracted from NB models built using consensus voting (>75% agreement) for** ***desirable*** **features.** Compounds selected by >75% of the chemists were categorized as desirable, and all others were categorized as undesirable. The 50 features most indicative of the desirable category that were present at least 2 times are reported in SMILES format.
(XLS)

Table S12 **ECFP4 features extracted from NB models built using consensus voting (>75% agreement) for** ***undesirable*** **features.** Compounds unselected by >75% of the chemists were categorized as undesirable, and all others were categorized as desirable. The 50 features most indicative of the undesirable category that were present at least 2 times are reported in SMILES format.
(XLS)
