# Supplementary figures and images for: Inside the Mind of a Medicinal Chemist: The Role of Human Bias in Compound Prioritization during Drug Discovery
Source: PLoS One. 2012 Nov 21;7(11):e48476. doi: 10.1371/journal.pone.0048476 (PMC3504051; doi:10.1371/journal.pone.0048476)

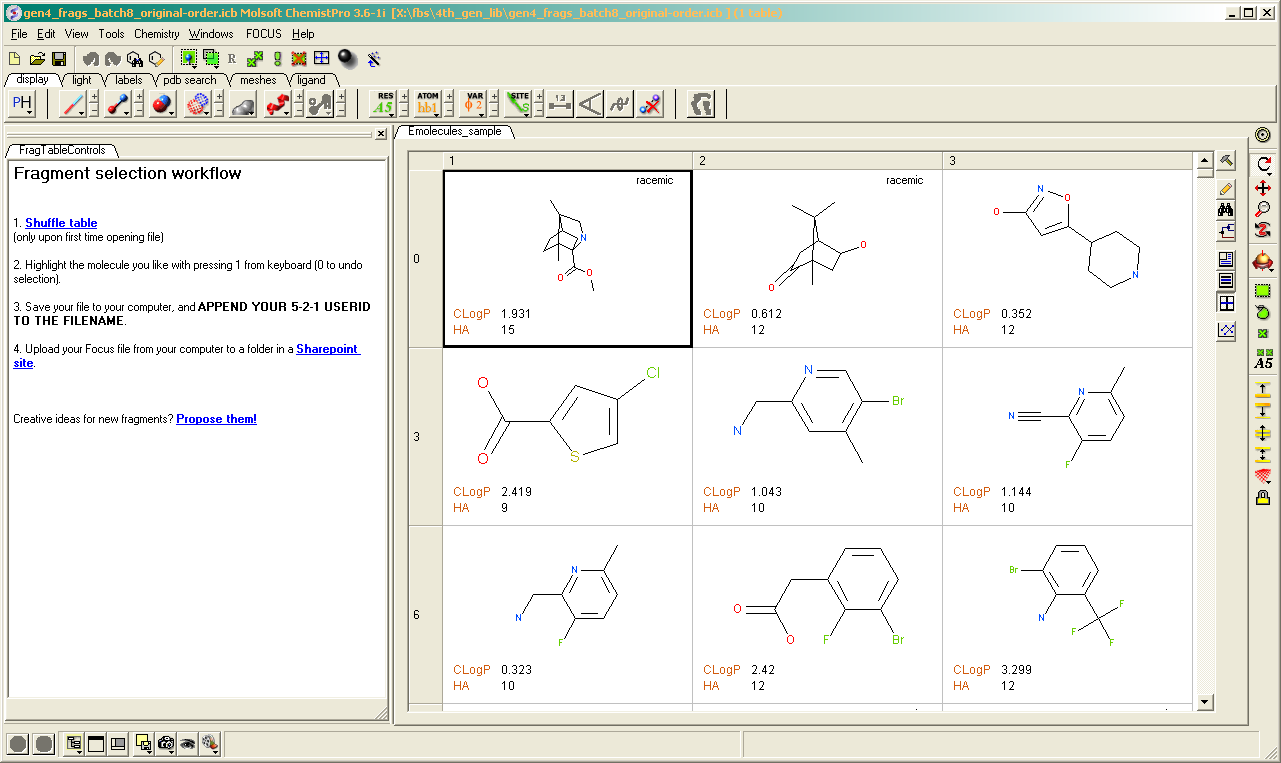

Supplement: Figure S1 — Simulated fragment selection session. (BMP) [file pone.0048476.s001.bmp]

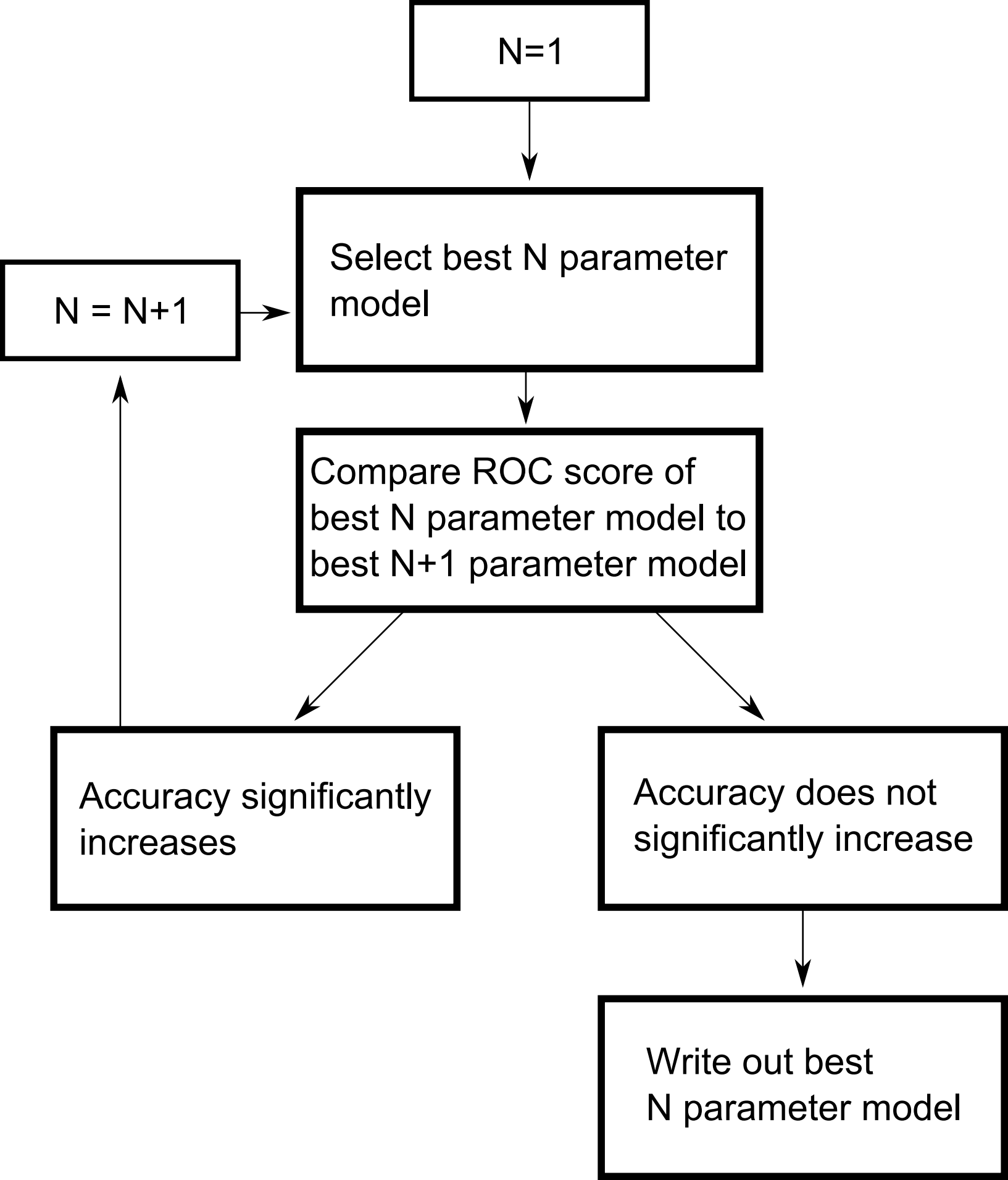

Supplement: Figure S3 — Feature subset selection for SNB classifiers. N is set to 1, and the best N parameter model is selected. It is then compared to the best N+1 parameter model. If the ROC score of the best N+1 parameter model is significantly more accurate than the current best N parameter model (difference>0.009), then N is incremented, and the process is repeated. If not (difference<0.009), then the current best N parameter model is selected. (PDF) [file pone.0048476.s003.pdf]

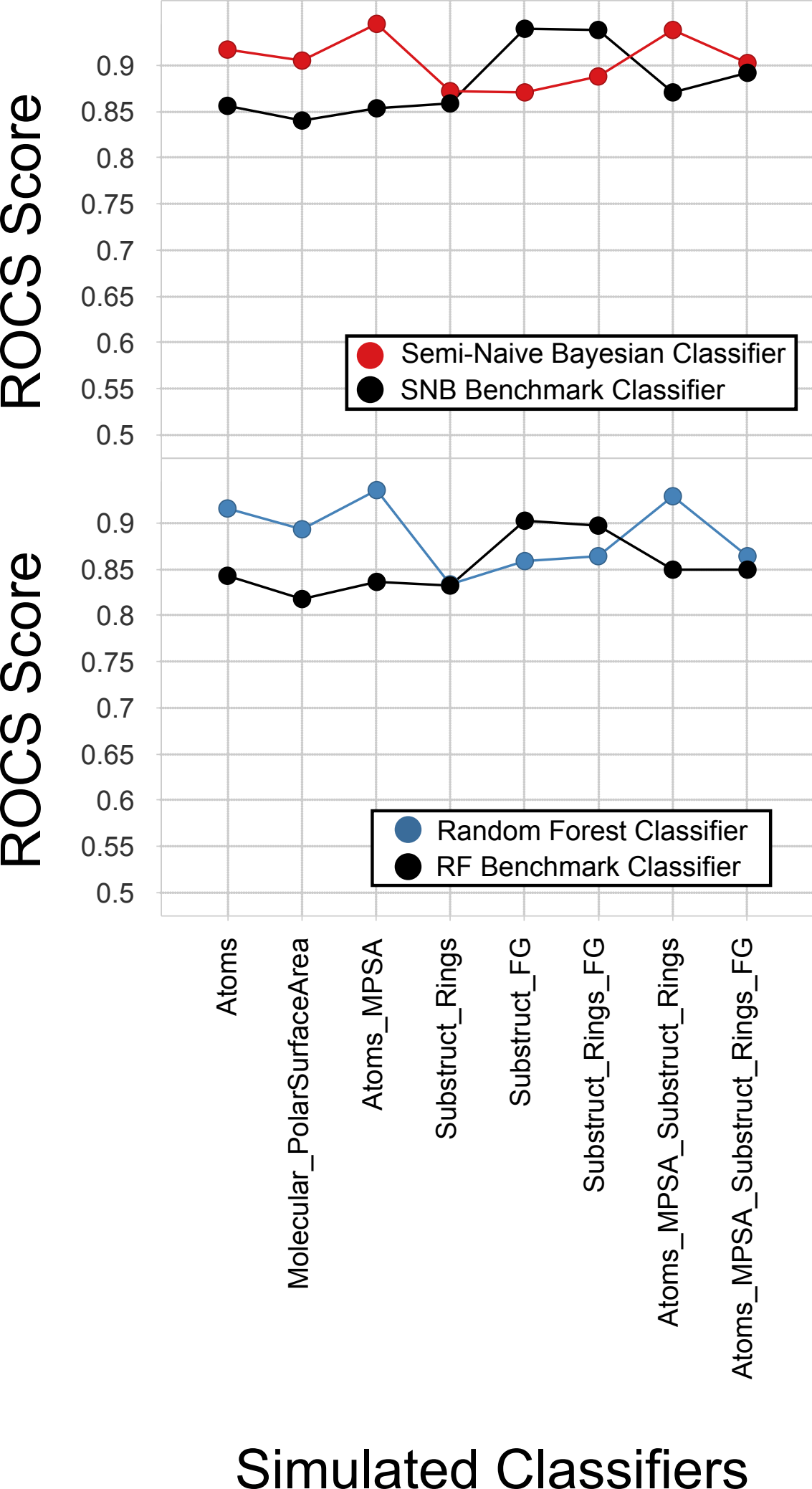

Supplement: Figure S4 — Predictive accuracies for SNB and RF classifiers when trained on selections made by simulated classifiers. (PDF) [file pone.0048476.s004.pdf]

# Simulated Classifiers

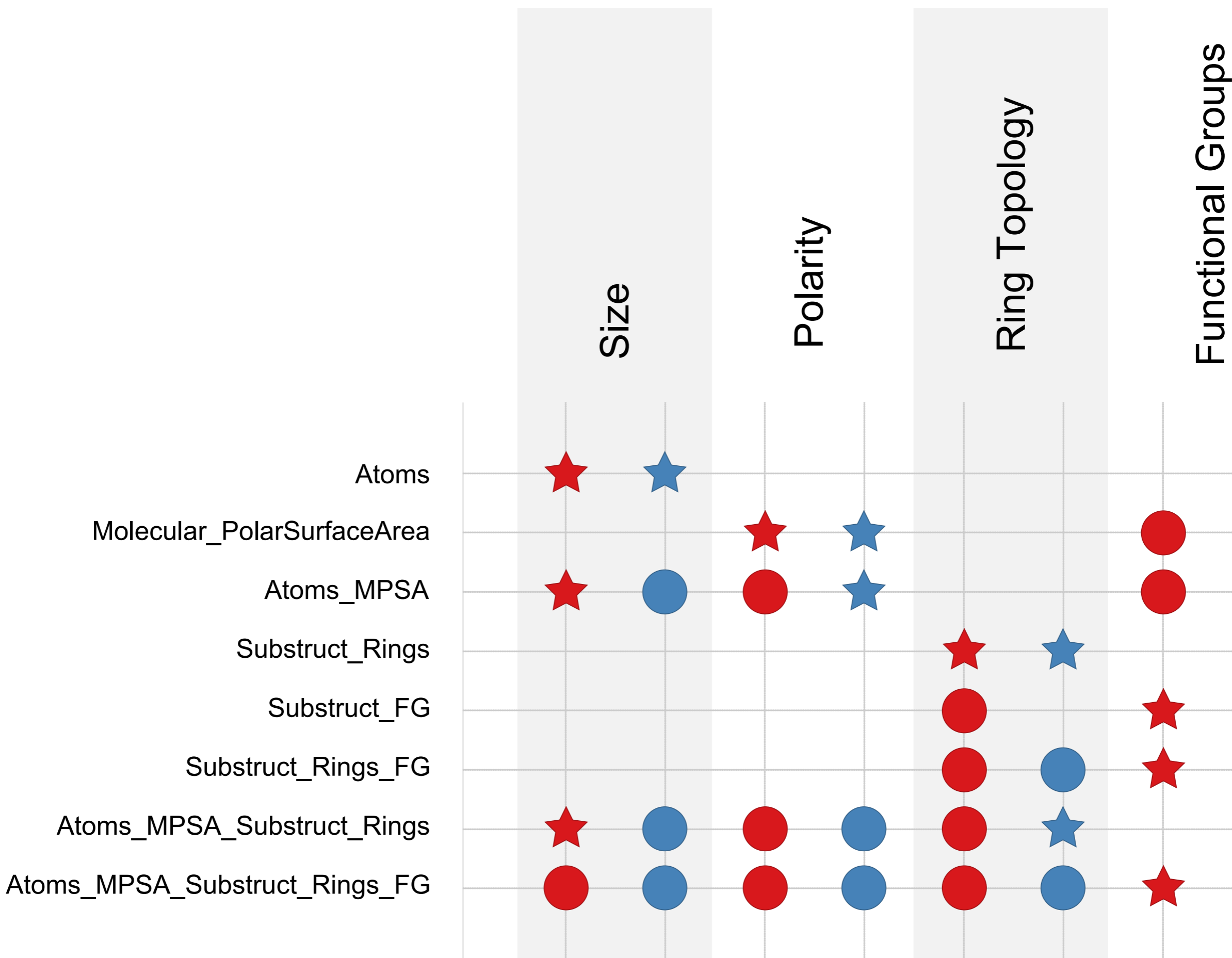

Supplement: Figure S5 — The parameters extracted from the SNB (red) and RF (blue) classifiers for selections made by simulated classifiers. The primary parameters for the classifiers are depicted as stars, and the secondary parameters are depicted as circles. (PDF) [file pone.0048476.s005.pdf]

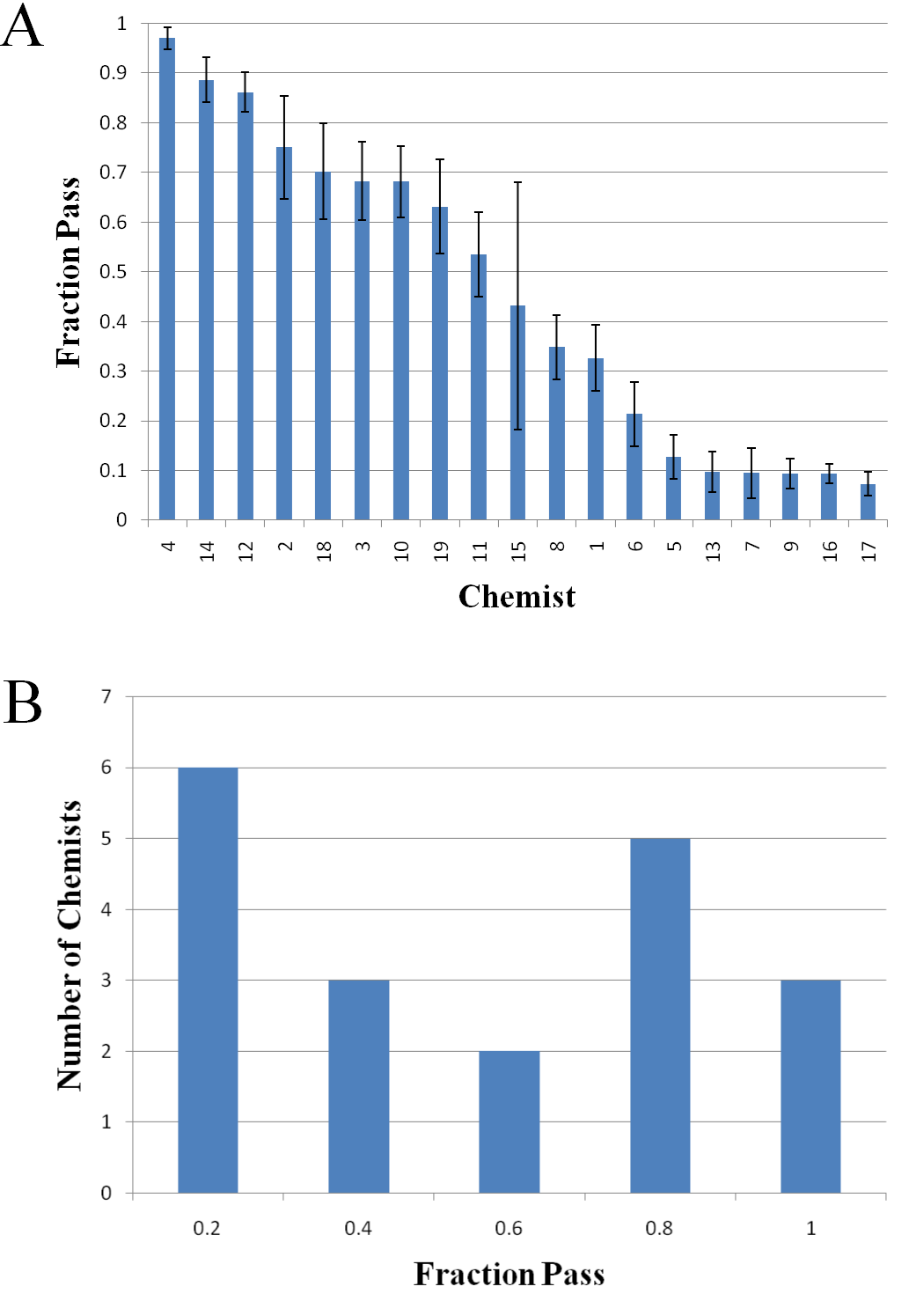

Supplement: Figure S6 — The fraction of compounds selected as desirable by each chemist. A: The fraction of compounds selected per batch by each chemist. The average fraction pass is 0.45 and the average standard deviation is 0.07. B: Histogram of the number of chemists that passed a specified fraction of fragments per batch. (PNG) [file pone.0048476.s006.png]

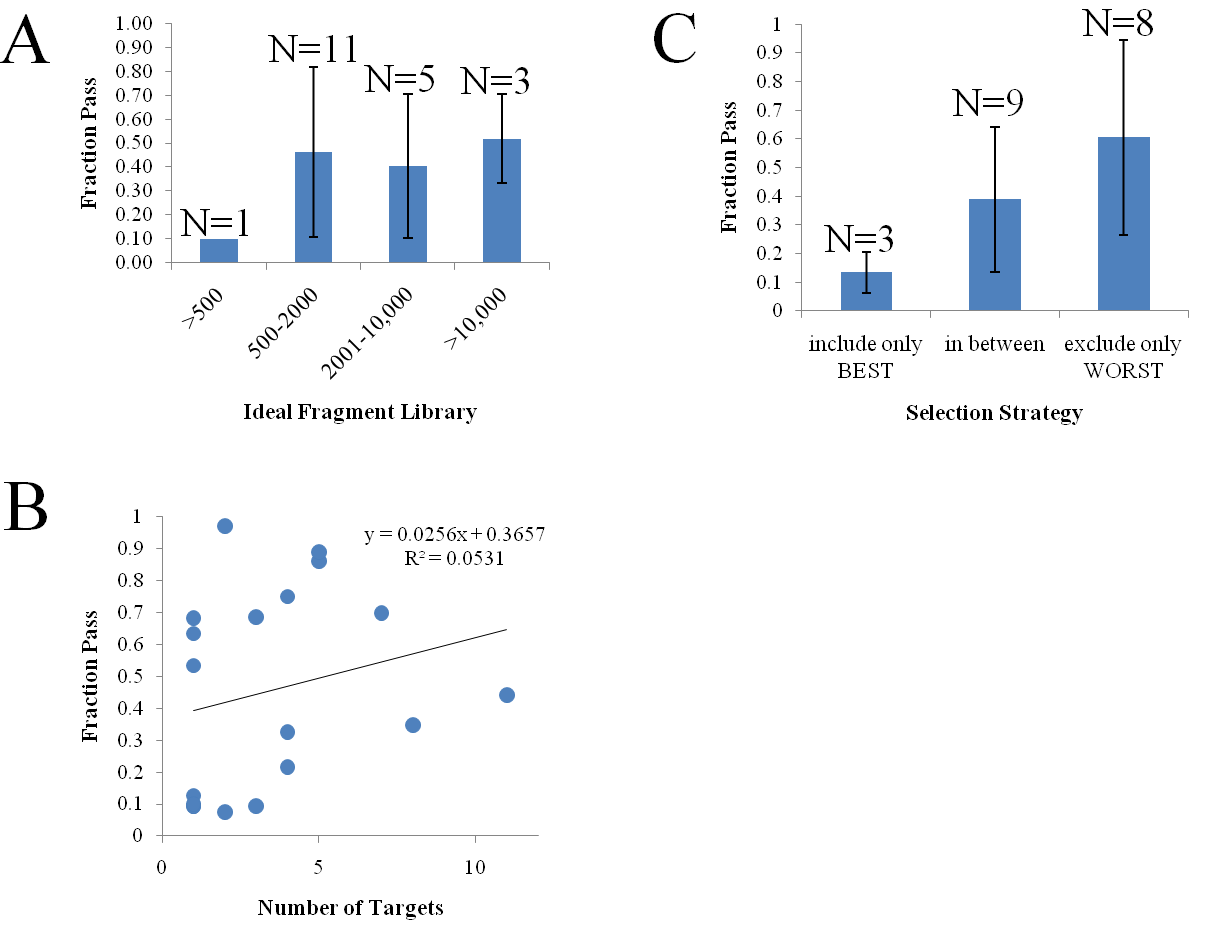

Supplement: Figure S7 — Relating the fraction of compounds selected as desirable to various factors. A: The average fraction of compounds passed per batch for chemists with different ideal fragment library sizes. B: The fraction of compounds passed versus the number of targets a chemist had worked on. C: The average fraction of compounds passed per batch for chemists with different selection strategies. Self-reports were used to obtain the ideal fragment size, number of past targets, and selection strategies. (PNG) [file pone.0048476.s007.png]

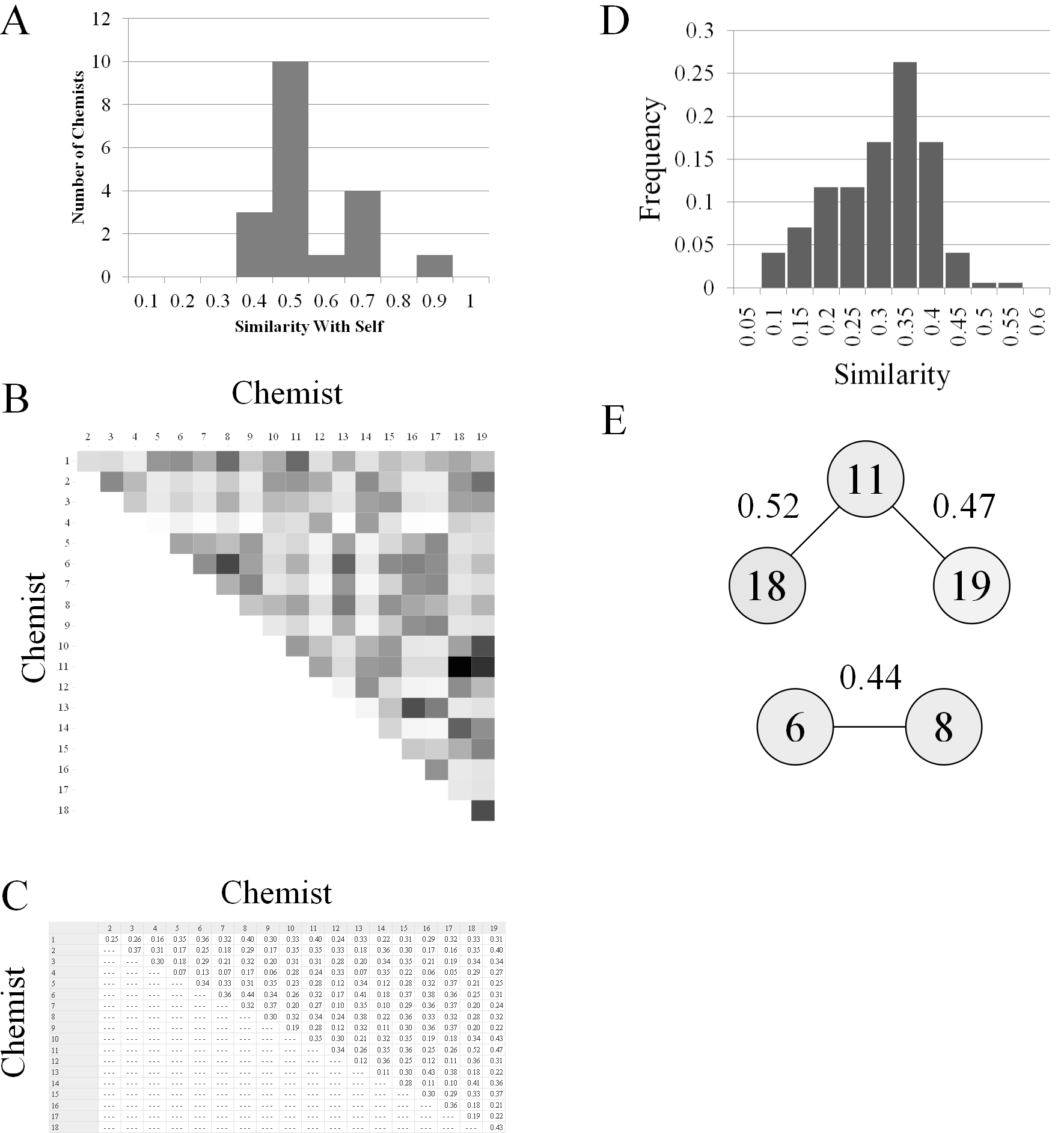

Supplement: Figure S8 — The similarity of selections when comparing chemists’ selections to themselves and to each other. A histogram of the modified Tanimoto similarities (S MT) comparing chemists to themselves (A). Similarities between chemists depicted as a heat map (B) and in table form (C). A histogram of modified Tanimoto similarities obtained between chemists (D). Two clusters formed by chemists using a modified Tanimoto similarity cutoff of ≥0.44 (E). (PNG) [file pone.0048476.s008.png]

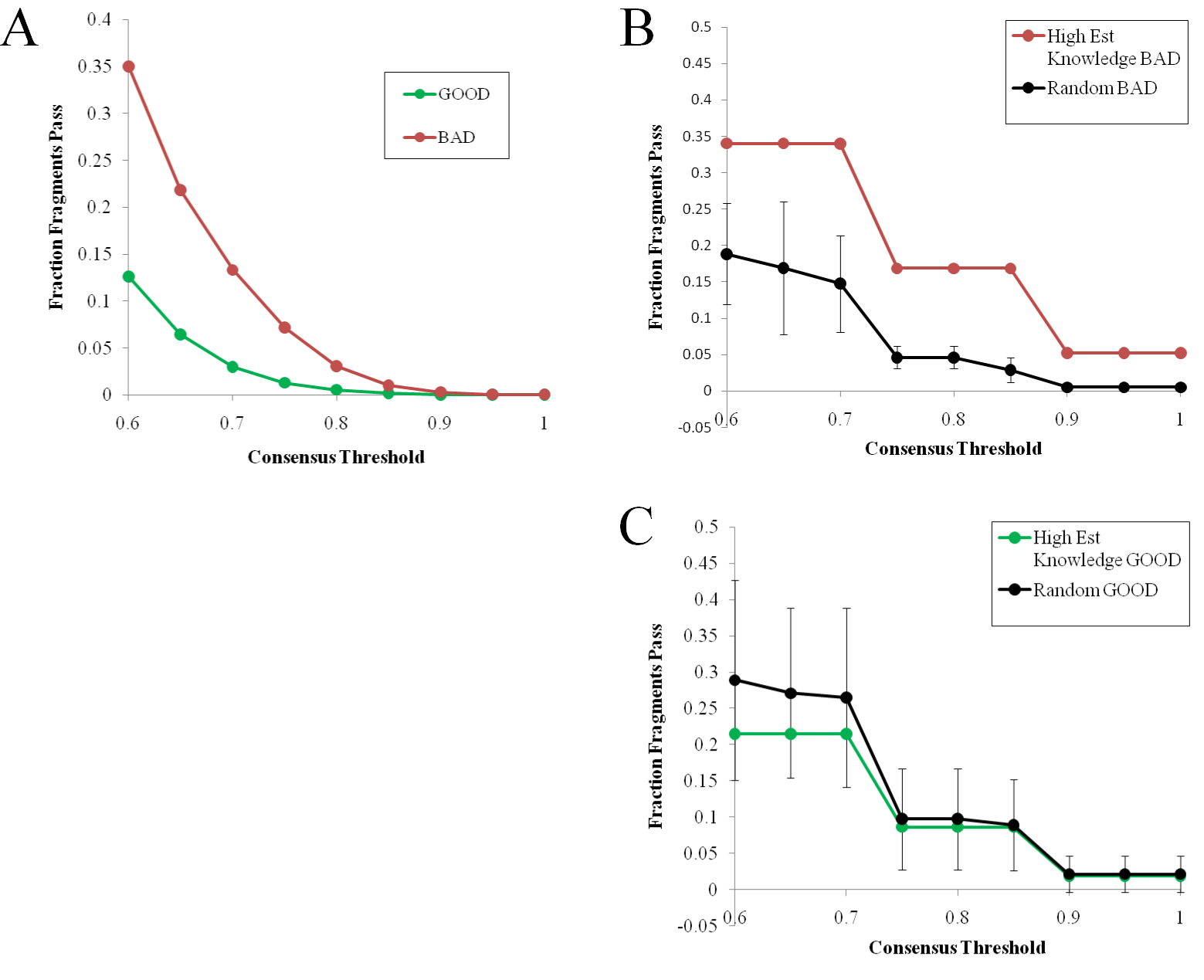

Supplement: Figure S9 — A comparison of consensus in desirable or undesirable fragments. A: The fraction of consensus good (green) or bad (red) compounds that pass when a given threshold for consensus is used. At all thresholds, there are more consensus good than consensus bad compounds. B: The fraction of consensus bad compounds for seven chemists with high estimated knowledge (red) versus seven randomly selected chemists (black) C: The fraction of consensus good compounds for seven chemists with high estimated knowledge (green) versus seven randomly selected chemists (black). (PNG) [file pone.0048476.s009.png]

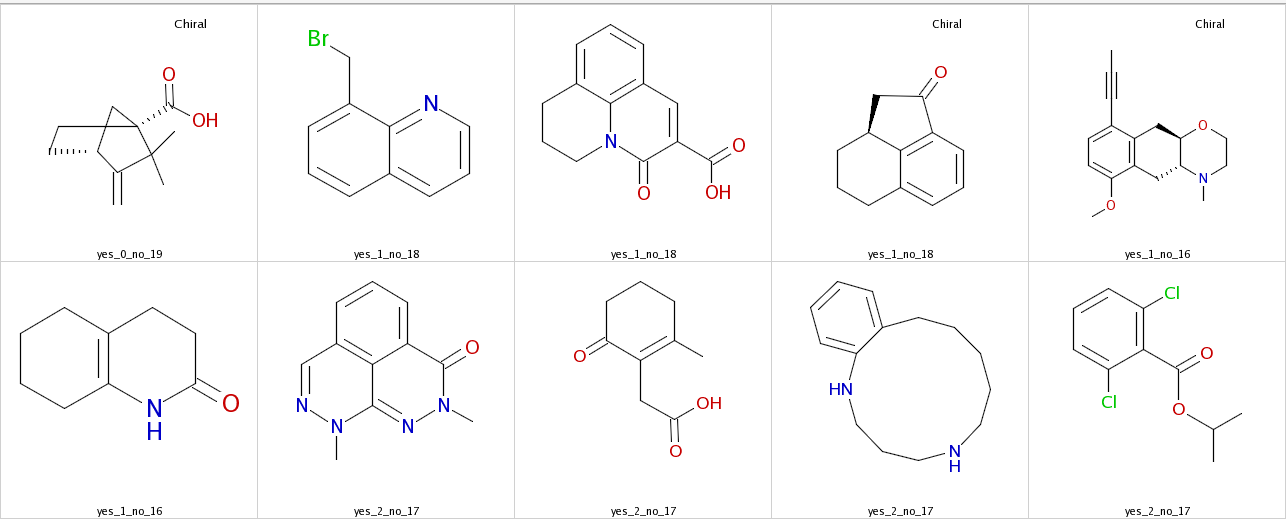

Supplement: Figure S10 — A selection of the fragments deemed worst by the group. The number of yes and no votes is below each structure. (TIF) [file pone.0048476.s010.tif]

# Chemists

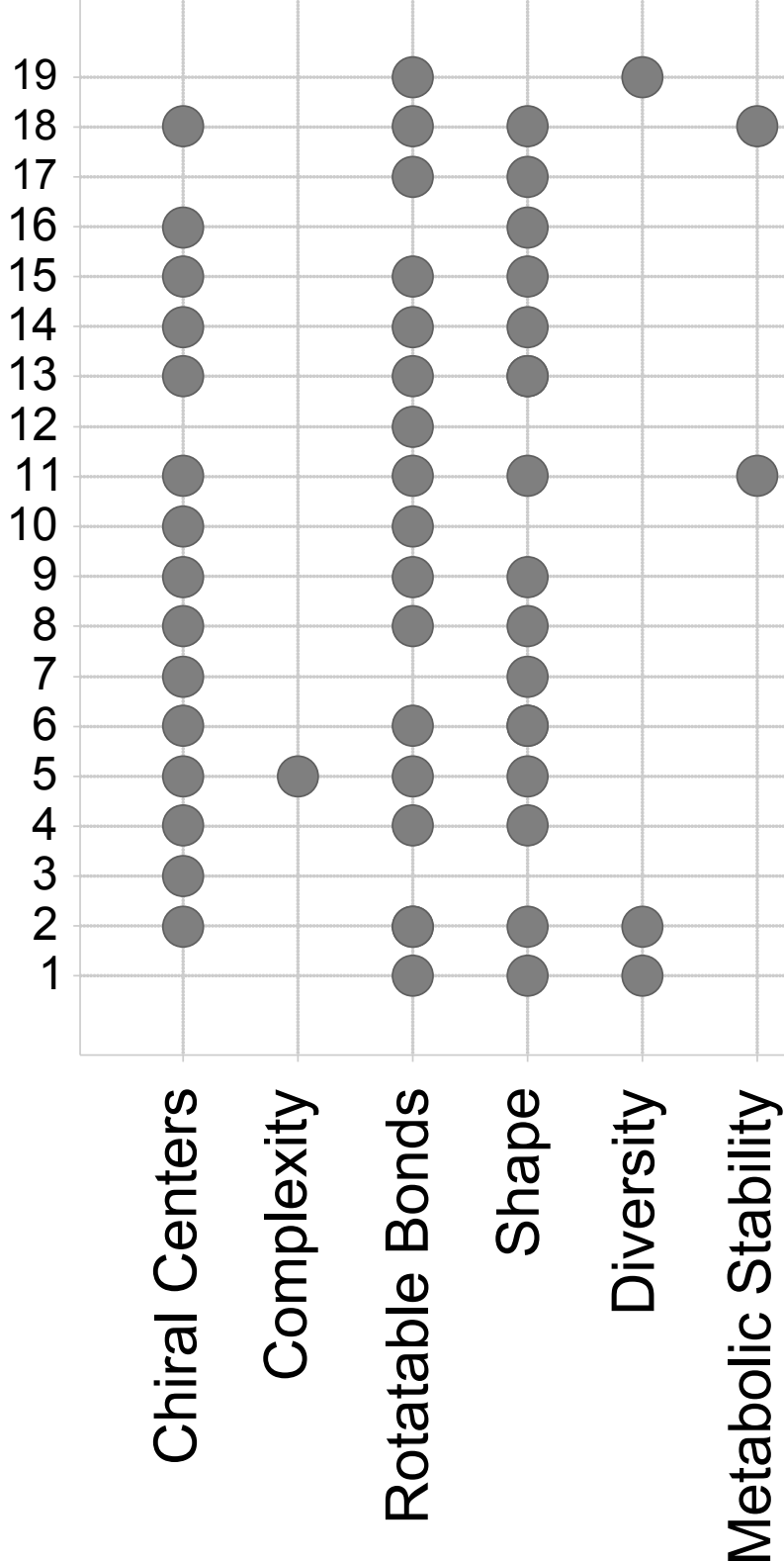

Supplement: Figure S11 — Parameters that were included in self-reports but not identified as important by SNB or RF models for each chemist. Note, “Diversity” and “Metabolic Stability” were self-reported, but attempts were not made to model these parameters. (PDF) [file pone.0048476.s011.pdf]
