# Supplementary material for: Inside the Mind of a Medicinal Chemist: The Role of Human Bias in Compound Prioritization during Drug Discovery
Source: PLoS One. 2012 Nov 21;7(11):e48476. doi: 10.1371/journal.pone.0048476 (PMC3504051; doi:10.1371/journal.pone.0048476)
Supplement: Figure S2 — A: Simulated classifiers selected fragments as good (green) or bad (red) based on thresholds for molecular polar surface area (MPSA) or number of atoms. The Bayes score of different bins for Naïve Bayesian models built using atoms (B), molecular polar surface area (C), atoms and molecular polar surface area independently (D), or atoms and molecular polar surface area jointly (E) are depicted. For the exclusive or (XOR) case (fourth panel in all rows), only the semi-naïve Bayesian model can correctly represent the simulated classifiers pattern. The ROCS score for each of the models is reported in corresponding panel for that model. The panel of the classification model that would be selected by the feature subset selection method that was employed is boxed with a black square. (PDF) [file pone.0048476.s002.pdf]

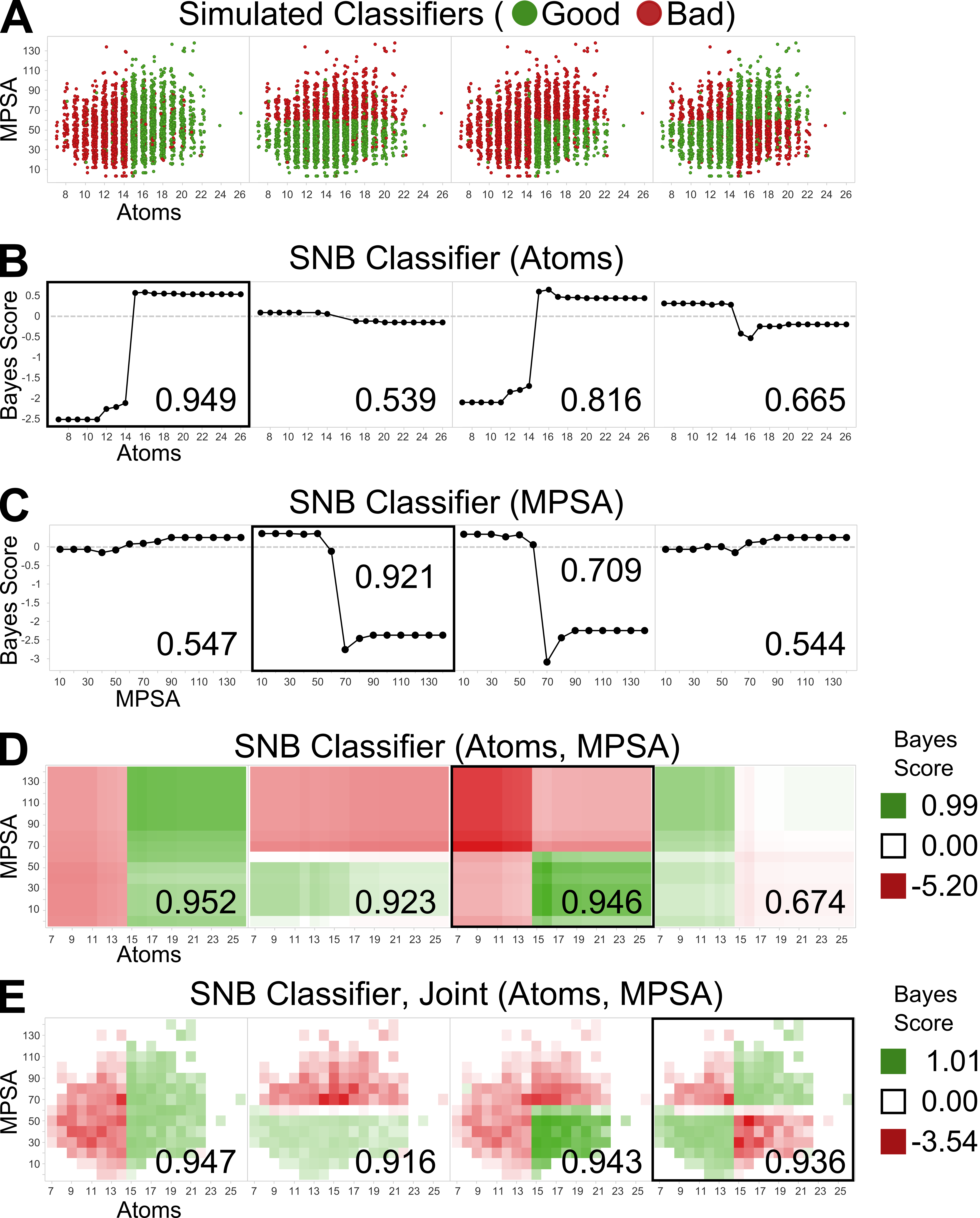

A
Simulated Classifiers (Good Bad)
MPSA
Atoms
B
SNB Classifier (Atoms)
Bayes Score
0.949
0.539
0.816
0.665
Atoms
C
SNB Classifier (MPSA)
Bayes Score
0.547
0.921
0.709
0.544
MPSA
D
SNB Classifier (Atoms, MPSA)
MPSA
0.952
0.923
0.946
0.674
Atoms
Bayes Score
0.99
0.00
-5.20
E
SNB Classifier, Joint (Atoms, MPSA)
MPSA
0.947
0.916
0.943
0.936
Atoms
Bayes Score
1.01
0.00
-3.54
