# Supplementary material for: Inside the Mind of a Medicinal Chemist: The Role of Human Bias in Compound Prioritization during Drug Discovery
Source: PLoS One. 2012 Nov 21;7(11):e48476. doi: 10.1371/journal.pone.0048476 (PMC3504051; doi:10.1371/journal.pone.0048476)
Supplement: Table S1 — Simulated classifiers with 1–4 rules for identifying good fragments are listed. (DOC) [file pone.0048476.s013.doc]

|  |  | Simulated Classifiers | | | | | | | |
| --- | --- | --- | --- | --- | --- | --- | --- | --- | --- |
|  |  | Atoms | Molecular_PolarSurfaceArea | Atoms_MPSA | Substruct_Rings | Substruct_FG | Substruct_Rings_FG | Atoms_MPSA_Substruct_Rings | Atoms_MPSA_Substruct_Rings_FG |
| Rules | Number atoms>13 | X |  | X |  |  |  | X | X |
|  | Molecular polar surface area ≤68.5 |  | X | X |  |  |  | X | X |
|  | No “bad” substructures: fused 6- and 6- or 6- and 5-membered aromatic rings |  |  |  | X |  | X | X | X |
|  | No “bad” substructures: nitro, ester, aliphatic bromine, 6- or 5-membered aromatic amine heterocycles |  |  |  |  | X | X |  | X |
