# Supplementary material for: Inside the Mind of a Medicinal Chemist: The Role of Human Bias in Compound Prioritization during Drug Discovery
Source: PLoS One. 2012 Nov 21;7(11):e48476. doi: 10.1371/journal.pone.0048476 (PMC3504051; doi:10.1371/journal.pone.0048476)
Supplement: Table S2 — Training and Test sets for 4-fold cross validation. The eight batches of compounds that were surveyed were jackknifed as follows to yield 4 training and test sets. (DOC) [file pone.0048476.s014.doc]

| Training/Test Set | Training  Batches | Test  Batches |
| --- | --- | --- |
| 1 | 3,4,5,6,7,8 | 1,2 |
| 2 | 1,2,5,6,7,8 | 3,4 |
| 3 | 1,2,3,4,7,8 | 5,6 |
| 4 | 1,2,3,4,5,6 | 7,8 |
