# Supplementary material for: Inside the Mind of a Medicinal Chemist: The Role of Human Bias in Compound Prioritization during Drug Discovery
Source: PLoS One. 2012 Nov 21;7(11):e48476. doi: 10.1371/journal.pone.0048476 (PMC3504051; doi:10.1371/journal.pone.0048476)
Supplement: Table S3 — Descriptors (72) used for building minimal Bayesian models. The parameter(s) that the descriptor is subsumed by is reported, as well as whether it was calculated using Pipeline Pilot (PP) or RDKit. Some descriptors were derived from combining or mathematically manipulating metrics previously calculated by Pipeline Pilot or RDKit (Custom). (DOC) [file pone.0048476.s015.doc]

| Parameter | Descriptor | Description | PP | RDKit | Custom |
| --- | --- | --- | --- | --- | --- |
| Size | Num_Atoms | Number of atoms | X |  |  |
| Size | Molecular_Weight | Molecular Weight | X |  |  |
| Size | atoms_bin | Number of atoms binned (5 atoms/bin) | X |  | X |
| Size | SP | Number of sp atoms |  | X |  |
| Size | SP2 | Number of sp^2^ atoms |  | X |  |
| Size | SP3 | Number of sp^3^ atoms |  | X |  |
| Size | SP_SP2_SP3 | Key containing the number of sp, sp^2^, and sp^3^ atoms |  | X | X |
| Size | scaffold_atoms | Number of atoms contained in Murcko scaffold | X |  | X |
| Shape | SP3_Fraction | Number of sp^3^ atoms divided by total atoms | X |  | X |
| Shape | Std_3 | Standard dimension of 3 (calculated in MOE) |  |  |  |
| Stereocenters | Num_StereoAtoms | Number of stereocenters | X |  |  |
| Rotatable Bonds | Num_RotatableBonds | Number of rotatable bonds | X |  |  |
| Polarity | Molecular_PolarSurfaceArea | Molecular polar surface area | X |  |  |
| Polarity | Molecular_FractionalPolarSurfaceArea | The molecular fraction of polar surface area | X |  |  |
| Polarity | RGroup_MPSA | Total polar surface area of R-Groups | X |  | X |
| Polarity | scaffold_MPSA | Total polar surface area of Murcko scaffold | X |  | X |
| Lipophilicity | ALogP | ALogP as computed in PP | X |  |  |
| Charge | Num_PositiveAtoms | Number of positive atoms | X |  |  |
| Charge | Num_NegativeAtoms | Number of negative atoms | X |  |  |
| Charge | ChargedAtoms | Total number of charged atoms | X |  | X |
| Charge | PosAtoms_NegAtoms | Key containing the number of positive and negative atoms | X |  | X |
| H-Bonding Groups | Num_H_Donors | Number of hydrogen bond donors | X |  |  |
| H-Bonding Groups | Num_H_Acceptors | Number of hydrogen bond acceptors | X |  |  |
| H-Bonding Groups | HB_total | Total number of hydrogen bonding groups | X |  | X |
| H-Bonding Groups | NPlusO_Count | Total number of nitrogens and oxygens | X |  |  |
| H-Bonding Groups | donors_acceptors_bin | Key containing the number of H-bond donors and acceptors, binned in intervals of 5 | X |  | X |
| H-Bonding Groups | RGroup_HBD | Number of hydrogen bond donors on R-Groups | X |  | X |
| H-Bonding Groups | RGroup_HBA | Number of hydrogen bond acceptors on R-Groups | X |  | X |
| H-Bonding Groups | Num_RGroups_HBDorHBA | Number of R-Groups that contain either a hydrogen bond donor or acceptor | X |  | X |
| H-Bonding Groups | scaffold_HBD | Number of hydrogen bond donors on Murcko scaffold | X |  | X |
| H-Bonding Groups | scaffold_HBA | Number of hydrogen bond acceptors on Murcko scaffold | X |  | X |
| Functional Groups | chemical_handles | Number of chemical handles (see table S3) | X |  | X |
| Functional Groups | Functional_Group_Key | Key with presence 1 or absence 0 of functional groups (see table S4) | X |  | X |
| Functional Groups | michael_acceptor | Number of michael acceptors (table S4) | X |  | X |
| Functional Groups | aliphatic_halogen | Number of aliphatic halogens (table S4) | X |  | X |
| Functional Groups | nitro | Number of nitro groups (see table S4) | X |  | X |
| Functional Groups | aniline_amine_prim_or_sec | Number of primary or secondary aniline amines (see table S4) | X |  | X |
| Chains | Num_Chains | Number of chains as computed in PP | X |  |  |
| Chains | Num_ChainAssemblies | Number of chain assemblies as computed in PP | X |  |  |
| Chains | C_CA | Key containing the number of chains and chain assemblies | X |  | X |
| Chains | Num_RGroups | Number of R-groups attached to the Murcko scaffold | X |  | X |
| Chains | RGroup_Atoms_total | Number of atoms defined by all R-groups | X |  | X |
| Chains | NSP | Number of non-ring sp atoms |  | X |  |
| Chains | NSP2 | Number of non-ring sp^2^ atoms |  | X |  |
| Chains | NSP3 | Number of non-ring sp^3^ atoms |  | X |  |
| Chains | NSP_NSP2_NSP3 | Key containing the number of non-ring sp, sp^2^, and sp^3^ atoms. |  | X | X |
| Chains | NSP3_fraction | The number of non-ring sp^3^ atoms divided by all non-ring atoms |  | X | X |
| Ring Topology | Num_Rings (NR) | Number of rings | X |  |  |
| Ring Topology | Num_AromaticRings | Number of aromatic rings | X |  |  |
| Ring Topology | Num_RingBonds (RB) | Number of ring bonds | X |  |  |
| Ring Topology | Num_AromaticBonds (AB) | Number of aromatic bonds | X |  |  |
| Ring Topology | Num_BridgeBonds | Number of bridge bonds | X |  |  |
| Ring Topology | Num_SpiroAtoms (SA) | Number of spiro atoms | X |  |  |
| Ring Topology | Num_RingAssemblies (RA) | Number of ring assemblies | X |  |  |
| Ring Topology | RB_AB | Key containing the number of ring and aromatic bonds | X |  | X |
| Ring Topology | RB_AB_RA | Key containing the number of ring bonds, aromatic bonds, and ring assemblies | X |  | X |
| Ring Topology | RB_AB_RA_NR | Key containing the number of ring bonds, aromatic bonds, ring assemblies, and number of rings | X |  | X |
| Ring Topology | RB_AB_RA_SA | Key containing the number of ring bonds, aromatic bonds, ring assemblies, and spiro atoms | X |  | X |
| Ring Topology | RB_AB_RA_NR_SA | Key containing the number of ring bonds, aromatic bonds, ring assemblies, and number of rings, and spiro atoms | X |  | X |
| Ring Topology | RSP | Number of ring sp atoms |  | X |  |
| Ring Topology | RSP2 | Number of ring sp^2^ atoms |  | X |  |
| Ring Topology | RSP3 | Number of ring sp^3^ atoms |  | X |  |
| Ring Topology | RSP_RSP2_RSP3 | Key containing the number of ring sp, sp^2^, and sp^3^ atoms. |  | X | X |
| Ring Topology | RSP_RSP2_RSP3_RA | Key containing the number of ring sp atoms, ring sp^2^ atoms, ring sp^3^ atoms, and ring assemblies. | X | X | X |
| Ring Topology | RSP3_fraction | The number of ring sp^3^ atoms divided by all ring atoms |  | X | X |
| Complexity | FCFP4_density | Number of FCFP4 substructures divided by the number of atoms | X |  | X |
| IP/Novelty | drug_sim | Tanimoto similarity to known drugs | X |  |  |
| Synthetic Accessibility | SAScore | Synthetic Accessibility Score of Ertl and Schuffenhauer(3) | X |  | X |
| Size, Rotatable Bonds | rbonds_atoms_bin | Key containing the number of rotatable bonds, and atoms, where atoms are binned at intervals of 5 | X |  | X |
| Size, Polarity | atoms_MPSA | Key containing the number of atoms and Molecular_PolarSurfaceArea, where the Molecular_PolarSurfaceArea is binned | X |  | X |
| Size, Ring Topology | rings_atoms_bin | Key containing the number of rings, and atoms, where atoms are binned at intervals of 5 | X |  | X |
| Size, Polarity, Ring Topology | atoms_MPSA_RSP_RSP2_RSP3_RA | Key containing the number of atoms, Molecular_PolarSurfaceArea, ring sp atoms, ring sp^2^ atoms, ring sp^3^ atoms, and ring assemblies, where then number of atoms and Molecular_PolarSurfaceArea are binned. | X | X | X |
