# Supplementary material for: Inside the Mind of a Medicinal Chemist: The Role of Human Bias in Compound Prioritization during Drug Discovery
Source: PLoS One. 2012 Nov 21;7(11):e48476. doi: 10.1371/journal.pone.0048476 (PMC3504051; doi:10.1371/journal.pone.0048476)
Supplement: Table S4 — Chemical handles. For the chemical_handles descriptor, chemical handles that a chemist might manipulate were counted. Specific types of substructures were only considered chemical handles if they were located on the core, on an R-group, or both. (DOC) [file pone.0048476.s016.doc]

| Handle | Core | R-Group | Both |
| --- | --- | --- | --- |
| Primary or Secondary Amine |  |  | X |
| Amide, non-ring |  | X |  |
| Amide, ring | X |  |  |
| Aromatic Bromine or Chlorine | X |  |  |
| Carboxylic Acid |  | X |  |
| Ester |  | X |  |
