# Supplementary material for: Inside the Mind of a Medicinal Chemist: The Role of Human Bias in Compound Prioritization during Drug Discovery
Source: PLoS One. 2012 Nov 21;7(11):e48476. doi: 10.1371/journal.pone.0048476 (PMC3504051; doi:10.1371/journal.pone.0048476)
Supplement: Table S5 — Functional groups included in functional group key. A fingerprint of medicinal chemistry relevant functional groups (smarts_fp) was developed to characterize the functional groups present or absent in a compound. SMARTS substructures were used to identify the presence of substructures, and these were combined into a functional group key. If the functional group is present in the fragment, the value for it in the key is 1, while if it is absent the value is 0. This is the descriptor used in the model illustrated for chemist 1 in Figure 4. (DOC) [file pone.0048476.s017.doc]

| Alkyne | Ester | Aniline Amine (1° or 2°) | Ketone |
| --- | --- | --- | --- |
| Alkene (non ring) | Carboxylic Acid | Amine (1° or 2°) | Sulfonamide |
| Michael acceptor | Aromatic Halogen | Amine (3°) | Sulfone |
| Phenol | Aliphatic Halogen | Amide | Urea |
| Hydroxyl | Fluorine | Imide | Aromatic Heteroatom |
| Ether | Trifluoromethyl | Nitro |  |
