# Supplementary material for: Inside the Mind of a Medicinal Chemist: The Role of Human Bias in Compound Prioritization during Drug Discovery
Source: PLoS One. 2012 Nov 21;7(11):e48476. doi: 10.1371/journal.pone.0048476 (PMC3504051; doi:10.1371/journal.pone.0048476)
Supplement: Table S6 — ROC Scores obtained for random simulated classifiers that passed different fractions of compounds. (DOC) [file pone.0048476.s018.doc]

| Fraction Pass | ROC Score |
| --- | --- |
| 0.1 | 0.55 ± 0.02 |
| 0.5 | 0.51 ± 0.003 |
| 0.9 | 0.54 ± 0.02 |
